# Survey Validation and Optimization to Assess Public Awareness of Origin Protected Designation (PDO) Cheeses: Majorero and Palmero Cases

**DOI:** 10.3390/foods15162881

**Published:** 2026-08-18

**Authors:** Eva Boyer Bustamante, María del Rosario Fresno Baquero, Cecilio José Barba Capote, Luis Alberto Bermejo Asensio, Álvaro Déniz Mesa, Francisco Javier Navas González

**Affiliations:** 1 International Doctoral School in Agri-Food (eidA3), University of Cordoba, Rabanales University Campus, 14071 Cordoba, Spain; ebobuvet@gmail.com; 2 Unidad de Producción Animal, Pastos y Forrajes en Zonas Áridas y Subtropicales, Instituto Canario de Investigaciones Agrarias (ICIA), 38260 Santa Cruz de Tenerife, Spain; mfresno@icia.es; 3 Department of Animal Production, Faculty of Veterinary Medicine, University of Córdoba, 14071 Cordoba, Spain; cjbarba@uco.es; 4 Departamento de Ingeniería Agraria y del Medio Natural, Escuela Politécnica Superior de Ingeniería—Sección Agraria, Universidad de La Laguna (ULL), Carretera de Geneto no 2, 38200 San Cristóbal de La Laguna, Spain; lasensio@ull.edu.es (L.A.B.A.); deniz.coke.513@gmail.com (Á.D.M.); 5 Department of Genetics, Faculty of Veterinary Medicine, University of Córdoba, 14071 Cordoba, Spain

**Keywords:** consumer behavior, authenticity, food labeling, certification schemes, sensory perception

## Abstract

Protected Designation of Origin (PDO) products play a vital role in preserving cultural heritage, supporting rural economies, and promoting local breed biodiversity. However, few studies have validated instruments capable of assessing consumer knowledge, perceptions, and trust regarding PDO labels and authenticity cues. This study validated and optimized a survey instrument designed to evaluate consumer attitudes towards PDO cheeses, using Majorero and Palmero cheeses from the Canary Islands as case studies. A total of 539 valid questionnaires were collected before and after structured educational sessions. Cross-validated Canonical Discriminant Analysis techniques were employed to assess the reliability, discriminatory capacity, and predictive accuracy of survey items. Results revealed significant improvements in consumer knowledge and perceptions following the educational intervention. Participants demonstrated greater understanding of PDO regulations, increased confidence in sensory evaluation, and enhanced ability to identify authenticity cues. The discriminant model achieved a classification accuracy of 96.23% (AUC = 0.9925), confirming excellent predictive performance. Consumer attitudes were primarily influenced by territorial attachment, familiarity, and trust in certification rather than technical knowledge. These findings support educational and marketing strategies to strengthen origin labeling, improve consumer literacy, reinforce authenticity perceptions, and promote PDO products’ cultural and economic sustainability while providing a validated framework for future consumer research.

## 1. Introduction

Goat cheeses vividly illustrate the link between local breed biodiversity, cultural heritage, and rural livelihoods. Produced across diverse climates—from Mediterranean islands to Alpine and Atlantic mountain valleys—they reflect pastoral communities’ adaptive strategies and autochthonous breeds in countries such as Spain, France, Italy, Greece, Portugal, and Turkey, where goat husbandry and artisanal cheesemaking have deep historical roots. Unlike standardized dairy products, many goat cheeses retain artisanal methods, local microbial ecologies, and unique organoleptic profiles. As nutritional resources and cultural markers, they anchor collective identity and generate value-added opportunities for rural territories. Global food market expansion and rising demand for authenticity have heightened the need to protect and promote these products [1].

In the EU, the Protected Designation of Origin (PDO) scheme ensures all production stages occur within a defined region and that product traits reflect the terroir and tradition [2,3]. Of ~180 PDO cheeses, only a few are goat cheeses, yet they strongly symbolize biodiversity and traditional know-how [4]. Globally, goat-milk production exceeds 20 million tonnes annually—mainly in Asia, Africa, and Europe—explaining the wide distribution of traditional goat cheeses, despite most lacking formal PDO protection [5]. This imbalance highlights both cultural richness and opportunities for broader recognition and legal protection of regional varieties [6].

In Spain, seven cheeses currently hold PDO status. Anthropologically, the PDO label ensures that a product originates from a specific region, with characteristics shaped by natural factors (climate, soil) and human factors, including artisanal practices passed through generations. This safeguards cultural identity, local knowledge, and community traditions. For cheeses, PDO certification guarantees that milk and production strictly follow traditional practices, ensuring typicality, authenticity, and traceability [7,8], which strengthens consumer trust through production-chain transparency [9].

The Canary Islands were pioneers in Spanish PDO recognition, hosting the first certified cheeses: Majorero from Fuerteventura (1996) and Palmero from La Palma (2001) [8,10]. The archipelago not only adopted PDO certification early but is also Spain’s leading region in goat-cheese production and consumption. By 2014, it produced ~12,500 tonnes of goat cheese annually across 53 dairies, highlighting both the sector’s economic significance and its deep cultural embeddedness [11].

Majorero and Palmero cheeses are closely tied to their native goat breeds. Majorero is made mainly from Majorera goat milk—hardy, high-yielding, and native to Fuerteventura—with up to 15% Canary sheep milk allowed in matured cheeses. It has a compact yet creamy texture, tangy-spicy flavor, and a rind traditionally rubbed with paprika or olive oil [8]. Palmero, produced exclusively from Palmera goat milk, reflects adaptation to La Palma’s volcanic terrain, with a firm, elastic paste and, when smoked, a white-to-brown rind [10].

The manual *Sensory Analysis of Pressed-Paste Goat Cheeses: Majorero PDO and Palmero PDO Cheese* integrates historical context, composition, processing, and sensory protocols, guiding producers and tasting panels to preserve distinctive profiles and enhance market competitiveness within PDO standards [12].

**Figure 1:**
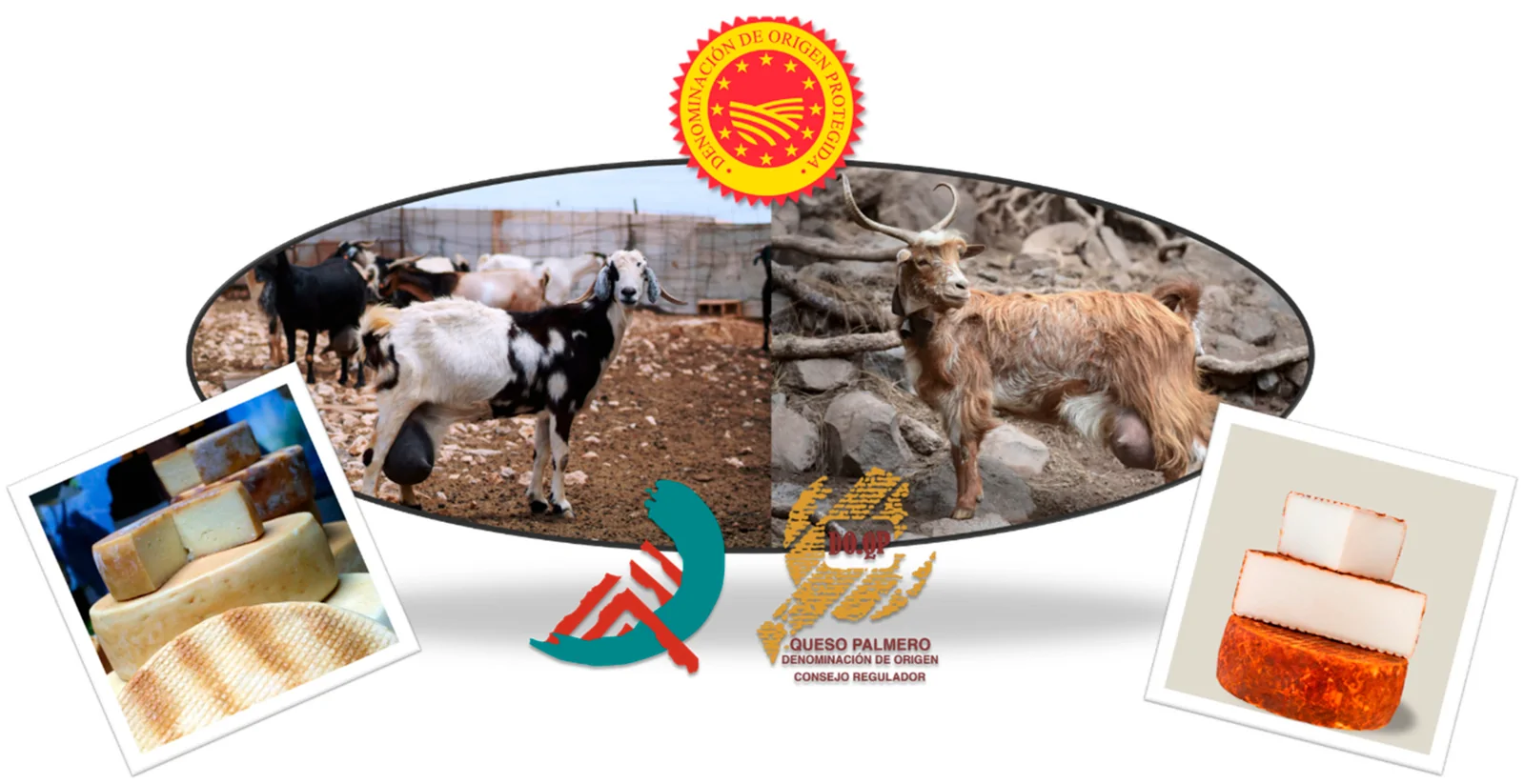
Majorera goat and Majorero Cheese with Logo Regulatory Council for Majorero Cheese (left) and Palmera goat and Palmero Cheese with Logo Regulatory Council for Palmero Cheese (right).

Institutional and genetic frameworks underpin both PDOs. The Majorera goat entered Spain’s Official Catalogue in 1981, with its herdbook managed by ANCAM since 1987; the Palmera goat was recognized in the 1990s, with its herdbook under ACP from 1999. These coordinated efforts led to PDO recognition—Majorero in 1996 and Palmero in 2001—ensuring breed integrity, authenticity, and traceability [13,14,15] (Figure 1).

Protected Designation of Origin (PDO) systems have become increasingly important within agri-food markets due to their capacity to preserve regional identity, protect traditional production systems, and enhance the commercial value of geographically linked foods. Previous research has shown that consumers frequently associate PDO labels with authenticity, artisanal production practices, traceability, heritage, and superior quality [16,17,18,19,20,21,22]. In traditional dairy products, particularly cheeses derived from autochthonous breeds, PDO certification also functions as a cultural and territorial marker capable of strengthening rural economies and preserving local biodiversity.

Consumer awareness and acceptance of PDO products are influenced by multiple interacting factors, including familiarity with certification systems, sensory perception, perceived authenticity, trust in labeling schemes, and willingness to pay premium prices [11,21,22,23,24,25]. However, despite the positive associations frequently attributed to PDO products, several studies have reported that limited understanding of certification labels, label proliferation, and price sensitivity may reduce consumer confidence and purchasing intention [23,24,26]. Consequently, legal certification alone does not necessarily guarantee market success or consumer trust.

Recent literature has additionally linked PDO-food perception with broader concepts of sustainable consumption, environmentally conscious purchasing behavior, and healthy food choices [27]. Studies addressing trust management in organic and certified food systems have emphasized that consumers increasingly evaluate food products not only according to sensory quality but also according to perceived environmental sustainability, production ethics, transparency, and health implications. Similarly, sustainable brand-management approaches highlight the importance of credibility, authenticity, and long-term trust-building in the valorization of traditional foods and regional products. These multidimensional aspects reinforce the need for robust analytical frameworks capable of accurately capturing the complexity of consumer perceptions toward PDO-certified foods.

Consumer-perception research in food science has traditionally relied on descriptive statistics, exploratory questionnaires, willingness-to-pay experiments, regression analyses, reliability coefficients, and factor-analysis approaches [11,25,26,28,29,30,31,32,33,34]. Although these methodologies have provided valuable insights into consumer preferences and market behavior, important methodological limitations remain regarding survey validation and predictive robustness.

One of the principal shortcomings identified in previous studies concerns the frequent reliance on resubstitution-based classification procedures, where model performance is evaluated using the same dataset employed to estimate the classification functions. Such approaches may artificially inflate classification accuracy and produce overly optimistic estimates of predictive performance, thereby increasing the risk of overfitting [1]. Similarly, many consumer-survey studies omit external validation or resampling procedures entirely, limiting the generalizability and reproducibility of their findings [2,3].

Another methodological limitation relates to the multidimensional structure of consumer-perception data. Variables such as trust, authenticity, sensory perception, familiarity, purchasing behavior, and cultural attachment are often highly interrelated and may interact simultaneously within heterogeneous consumer groups. Conventional regression-based approaches are frequently less effective in capturing these complex multivariate relationships because they are primarily designed to model isolated dependent outcomes rather than maximize discrimination among predefined groups [31,32].

Furthermore, although repeated consumer responses and clustered perception patterns may introduce within-subject or within-group correlations, these structures are rarely addressed explicitly in PDO-consumer research. Ignoring such dependencies may compromise the stability and transferability of classification models, particularly when moderate sample sizes and heterogeneous populations are involved. These limitations highlight the importance of incorporating robust validation procedures capable of improving model reproducibility and reducing risks of overfitting.

Within this context, Canonical Discriminant Analysis (CDA) offers important methodological advantages for the validation of consumer-perception surveys. CDA is specifically designed to identify linear combinations of variables that maximize between-group variance while minimizing within-group variance, thereby facilitating the classification of heterogeneous populations and the identification of the variables contributing most strongly to group separation [33,34,35,36,37,38,39,40]. In sensory and consumer studies, CDA has been successfully applied to distinguish consumer profiles, evaluate sensory-derived categories, and identify discriminating attributes associated with food preferences and purchasing behavior [1,4].

Compared with simpler analytical approaches, CDA simultaneously evaluates discriminatory capacity, variable contribution, and classification performance within a multivariate framework. This characteristic is particularly relevant in PDO-food research, where consumer responses frequently integrate sensory, cultural, behavioral, and trust-related dimensions. CDA therefore provides not only predictive capability but also interpretative value regarding the factors driving consumer differentiation.

The incorporation of cross-validation procedures further strengthens the methodological robustness of discriminant models. Techniques such as leave-one-out cross-validation (LOOCV), K-fold cross-validation, split-sample validation, and permutation testing generate out-of-sample error estimates that are substantially more reliable than resubstitution accuracy alone [2,3]. These procedures reduce the risk of overfitting and improve the generalizability, stability, and reproducibility of classification models, particularly in moderate and heterogeneous datasets commonly encountered in consumer research.

The combined application of CDA and cross-validation therefore addresses both explanatory and predictive objectives in survey validation. CDA identifies the multidimensional structure underlying consumer-group differentiation, whereas cross-validation evaluates the robustness and transferability of the resulting discriminant functions. Despite these advantages, studies integrating CDA-based validation frameworks with consumer-perception surveys for PDO-certified traditional foods remain scarce, particularly for products linked to autochthonous breeds and geographically specific production systems.

Accordingly, the aim of the present study was to validate and optimize a survey specifically tailored to the Majorero and Palmero PDO cheeses of the Canary Islands, ensuring that consumer perception is measured with both precision and reliability. Specifically, the study aimed to evaluate the discriminative capacity of questionnaire items related to PDO knowledge, trust, sensory perception, and purchasing behavior; identify the variables contributing most strongly to differentiation among consumer profiles; assess the robustness and predictive stability of the classification model through cross-validation procedures; and propose a replicable methodological framework for validating consumer-perception surveys applicable to other traditional foods derived from autochthonous breeds.

By addressing these objectives, the study contributes both theoretically and practically. Theoretically, it expands the methodological literature on PDO-food research by demonstrating the utility of CDA and cross-validation procedures for survey validation beyond conventional descriptive or regression-based analyses. Practically, it provides producers, policymakers, and marketers with a statistically validated tool for evaluating consumer perceptions and designing evidence-based communication and promotion strategies aimed at strengthening consumer trust and enhancing the sustainable valorization of traditional products.

## 2. Materials and Methods

### 2.1. Sampling Procedure

A non-probabilistic convenience sampling strategy was employed in the present study. Participants were recruited voluntarily from organized training activities conducted across the Canary Islands, including Fuerteventura, Tenerife, Gran Canaria, and La Palma. Recruitment involved educational institutions, professional associations, agricultural training centers, cultural organizations, and producer groups, ensuring the inclusion of participants from diverse sociodemographic and professional backgrounds. This approach enabled the collection of a heterogeneous sample composed of consumers, students, professionals, and cheese-sector stakeholders, which was considered appropriate for the exploratory and survey-validation objectives of the study. Nevertheless, because participation was voluntary and non-random, the findings should be interpreted with caution regarding their generalizability to the broader population.

### 2.2. Sample, Respondent Profile and Demographics

A total of 281 respondents attended the training sessions, generating 562 questionnaires (one pre-training and one post-training questionnaire per participant). One questionnaire was excluded because it was returned completely blank and contained no usable information, resulting in an initial dataset of 561 questionnaires from 281 respondents.

The dataset was subsequently subjected to a data-cleaning process to ensure the completeness and consistency of participants’ demographic information. During the first stage, questionnaires corresponding to six respondents were excluded because profession and/or age information was missing. This resulted in a dataset comprising 539 valid questionnaires from 270 participants who attended the training activities conducted in Fuerteventura, Tenerife, Gran Canaria, and La Palma.

In the second stage, questionnaires containing incomplete responses to the survey items were excluded. Following this quality-control procedure, the final dataset consisted of 530 questionnaires completed by 266 respondents, including 266 pre-training and 264 post-training questionnaires. The slight discrepancy between the number of pre- and post-training questionnaires resulted from two participants leaving the training session before its completion and therefore not completing the post-training questionnaire. These missing questionnaires represented only 0.38% of the total dataset and were treated as missing values. Given their negligible proportion and the group-level analytical framework adopted, this minimal loss of data did not affect the statistical analyses or compromise the robustness of the results. All questionnaires were administered under supervised conditions during the organized training sessions to minimize external distractions and maximize response completeness.

**Figure 2:**
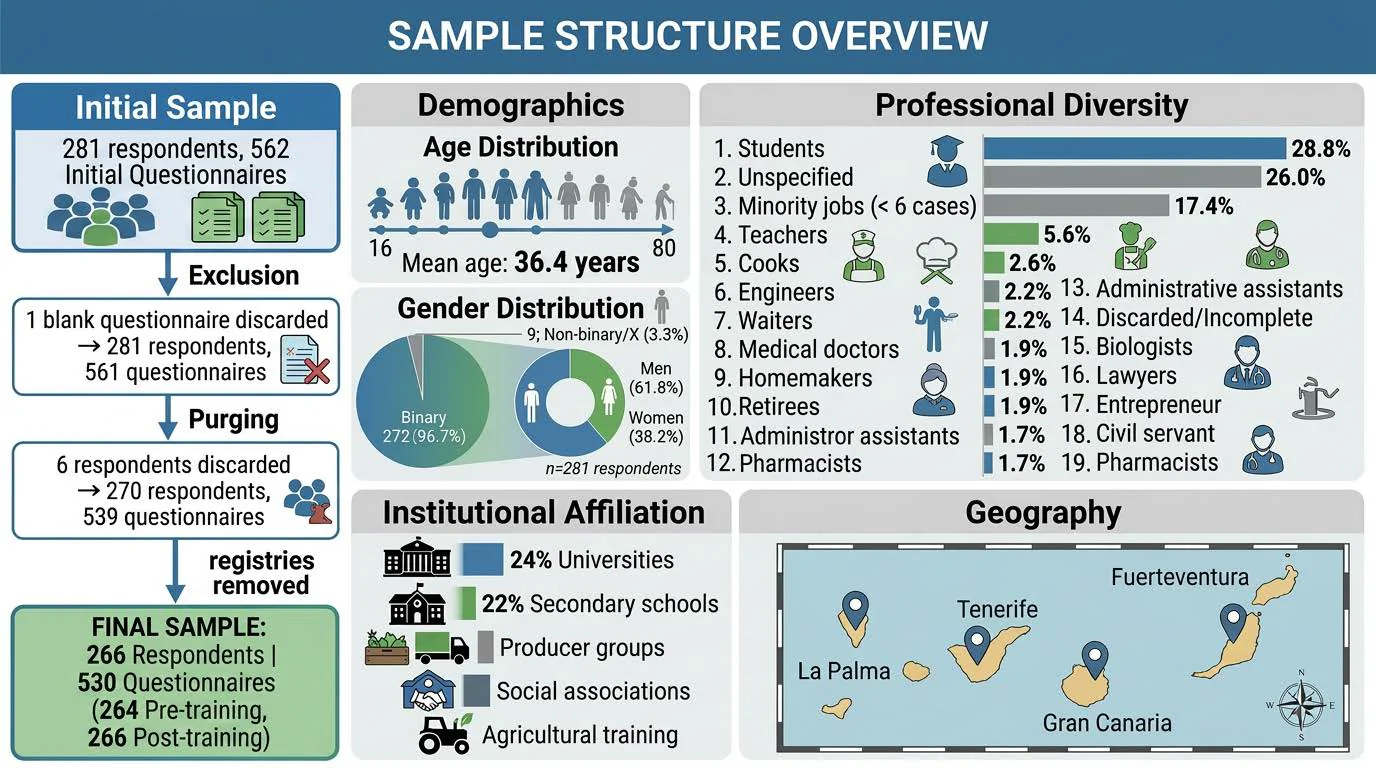
Overview of the study sample structure and participant characterization. The flow diagram summarizes the participant selection process, including initial recruitment, exclusion criteria, purging procedures, and the final validated sample used for analysis.

No further exclusion criteria were applied apart from registry incompleteness. All remaining questionnaires contained sufficient information to support the planned statistical analyses. The final sample was heterogeneous with respect to age, gender, profession, and institutional affiliation. The demographic, professional, institutional, and geographic characteristics of the study population are summarized in Figure 2 to provide a comprehensive description of the final sample.

• Age: Participants ranged in age from 16 to 80 years (mean ± SD: 36.4 ± 17.1 years). The inclusion of participants aged 16 years reflected the eligibility criteria for the organized training activities and was consistent with the applicable ethical procedures. Age was analyzed as a continuous quantitative variable to preserve statistical information and avoid arbitrary categorization. The age distribution was skewed toward younger participants, with 25% younger than 20 years, 50% younger than 32 years, and 75% younger than 52 years.
• Binary Gender: Initial sample comprised 333 questionnaires from 167 men (61.8%) and 210 questionnaires from 105 women (38.2%).
• Non-Binary Gender: 18 questionnaires from 9 respondents (3.3%) identifying as “X” or non-binary and 543 from 272 binary respondents (96.7%).
• Profession: Occupations were diverse, led by students (155; 28.8%), followed by teachers (30; 5.6%), cooks (14; 2.6%), engineers (12; 2.2%), waiter (12; 2.2%), medical doctors (10; 1.9%), homemakers (10; 1.9%), retirees (10; 1.9%), administrative assistants (10; 1.9%), biologists (9; 1.7%), lawyers (8; 1.5%), entrepreneur (8; 1.5%), civil servant (8; 1.5%) and pharmacists (8; 1.5%). A 17.4% was represented by minority job possibilities below 6 cases. In further 140 questionnaires (26%), respondents did not specify a profession, and 9 (1.7%) questionnaires were discarded due to the finding of incomplete registries.
• Recruitment setting: Participants were recruited through a broad range of educational institutions, professional organizations, and community groups across the Canary Islands. The largest proportions were recruited from secondary schools (22%) and universities (24%), followed by agricultural training centers (9%), cultural or social associations (11%), and producer or professional organizations (15%). Smaller proportions (<5%) were recruited from research institutes, local businesses, and specialized training courses. Recruitment was conducted across Fuerteventura, Tenerife, Gran Canaria, and La Palma, ensuring representation of both the general public and stakeholders associated with the cheese sector.

Overall, the survey encompassed a broad cross-section of consumers, students, professionals, and producers, enabling analysis of PDO Majorero and Palmero cheese perceptions across diverse sociodemographic backgrounds.

### 2.3. Survey Design and Structure

A structured questionnaire was developed to evaluate consumer knowledge, attitudes, and sensory self-assessment related to Canarian cheeses with Protected Designation of Origin (PDO), focusing on Majorero and Palmero. The instrument was designed within the framework of the project *RTA2014-00047-C-00-00: Valorization of traditional goat cheeses linked to an autochthonous breed* and administered to participants both before and after educational sessions.

The questionnaire contained 112 items in total, organized into five main sections:

1. Sociodemographic Information (6 items)
  ∘ Age, gender, profession, and a personal identifier code to ensure anonymity and data protection.
  ∘ Cheese preference (yes/no).
  ∘ Previous training in sensory analysis (yes/no), including an open field to specify the product(s).
2. General Knowledge and Training (5 items, including 1 open-ended)
  ∘ Self-assessed knowledge of Canarian cheeses, PDOs, and sensory analysis (3 items, each scored on a 3-point scale: low, medium, high).
  ∘ Previous training in sensory analysis (1 item, yes/no).
  ∘ Specification of product(s) evaluated in training (1 open response).
3. Sensory Perception and Self-Assessment (27 items). Respondents rated their ability to identify sensory parameters on a 5-point Likert scale (1 = very difficult, 5 = very easy).
  ∘ Visual and textural attributes (12 items): external appearance, appearance when cut, tactile texture, roughness, elasticity, humidity, texture in mouth, firmness, friability, oral humidity, adherence, solubility, and granulosity.
  ∘ Olfactory attributes (7 items): general aroma, intensity, lactic family, vegetal family, fruity family, roasted family, and spices family, with an open option for “other families.”
  ∘ Taste attributes (8 items): salty, sweet, acidic, bitter, spicy, astringent, aftertaste, and persistence, with an open option for “other tastes.”
4. Knowledge and Perceptions of PDO (69 items total)
  Statements were evaluated as true, false, or don’t know (NS).
  ∘ General PDO knowledge (29 items): addressed hygienic–sanitary guarantees, added value, sustainability, fraud prevention, territorial link, breed association, production rules, labeling, EU protection, permitted species, and producer/administration responsibilities.
  ∘ PDO Majorero (20 items): covered permitted breeds and herdbook standards, production zones, use of sheep milk, maturation practices, labeling requirements, counter-labels, coatings, use of coagulants, role of the breed, and producer registration.
  ∘ PDO Palmero (20 items): mirrored the Majorero block, addressing Palmera goat requirements, production zones, maturation practices, labeling, coatings, use of coagulants, role of the breed, and producer registration.
5. Labeling and Differentiation (7 items: 5 ranked + 2 open-ended)
  ∘ Ranking task (1–5) of factors that should differentiate PDO cheeses on labels: island, breed, feeding system, producer, and production method.
  ∘ Open fields for additional labeling elements (e.g., “other elements to be included”) and for final comments.

The questionnaire ended with an optional item inviting participants to provide their email addresses if they wished to receive updates on study results. The full survey instrument, including the original Spanish version and its English translation, is provided as Supplementary Material S1.

The questionnaire presented in Supplementary Material S1 was developed using three complementary sources of information: (i) the official Protected Designation of Origin (PDO) regulations for Queso Majorero and Queso Palmero, (ii) technical and sensory-analysis literature on traditional Canarian goat cheeses, and (iii) previous educational and dissemination materials focused on cheese sensory evaluation and PDO characteristics. The items were not selected arbitrarily but through a structured content-based approach designed to evaluate consumer knowledge, sensory perception, and understanding of PDO certification before and after the training intervention. Since the objective of the study was applied and exploratory rather than theory-testing, the questionnaire was not based on a single behavioral or psychometric theoretical framework. Instead, item selection was guided by the practical, sensory, and regulatory dimensions that define PDO cheeses, ensuring alignment with the educational objectives of the training sessions.

The sections related to PDO knowledge and regulatory compliance were specifically derived from the official regulations governing Queso Majorero and Queso Palmero, including aspects such as geographical production areas, authorized breeds, labeling requirements, maturation practices, and cheesemaking procedures [8,40]. In parallel, the sensory-analysis section of the questionnaire was structured according to descriptors and evaluation criteria described in the technical manual by Fresno Baquero and Álvarez Ríos [13], which details organoleptic attributes, texture descriptors, aroma families, and tasting methodologies used for traditional goat cheeses. Additional items related to participant demographics, previous training experience, and familiarity with PDO products were incorporated to contextualize consumer perceptions and training effects. Overall, the questionnaire integrated demographic variables, prior training experience, sensory perception, and PDO-related knowledge in order to evaluate changes before and after training sessions within the framework of the RTA2014-00047-C-00-00 project.

### 2.4. Survey Administration

The questionnaire was administered at two evaluation points to assess immediate changes in participants’ knowledge, perceptions, and self-assessed sensory competence following the educational intervention. A total of 266 questionnaires were completed immediately before the training sessions (pre-intervention assessment), establishing baseline information regarding PDO knowledge, sensory perception, and familiarity with Canarian cheeses. Following completion of the educational session, the same questionnaire was administered again to obtain post-intervention responses, yielding 264 completed questionnaires and enabling direct before–after comparisons within a standardized quasi-experimental framework.

Data collection was conducted in successive training rounds between October 2016 and March 2017. The first evaluation sessions took place on 31 October and 1 November 2016, followed by additional sessions during December 2016, February 2017, and March 2017. This phased implementation facilitated the inclusion of participants from diverse educational and professional backgrounds while maintaining identical survey structure, training content, and administration procedures across all groups.

To minimize potential information contamination between successive training rounds, each training session was conducted independently using an identical protocol. Participants completed the pre-training questionnaire before receiving any educational content and were explicitly instructed not to discuss the questionnaire or training materials with individuals who had not yet attended subsequent sessions. These measures were implemented to reduce the possibility of information transfer between participant groups while preserving the consistency of the educational intervention.

The slight discrepancy between the number of pre-training (*n* = 266) and post-training (*n* = 264) questionnaires was attributable to two participants who left the sessions before completion and therefore did not complete the post-intervention assessment. These cases were coded as missing values, representing 0.38% of the total dataset. Given the minimal proportion of missing data and the group-level analytical strategy employed, this marginal loss was not considered sufficient to affect the statistical analyses or compromise the robustness of the results.

Administration was conducted under supervised conditions within organized educational environments to minimize external distractions and maximize response completeness.

### 2.5. Survey Timing Rationale

Pre- and post-intervention questionnaires were administered immediately before and after each training session. Each session followed a standardized two-hour format. Although minor variations in total duration (±30 min) occurred depending on participant number and the length of discussion periods, the sequence, structure, and instructional content remained constant across sessions.

The short and standardized interval between assessments was intentionally selected to evaluate immediate learning effects and short-term perceptual changes directly attributable to the intervention, consistent with quasi-experimental pre–post educational designs [34]. This design minimized the influence of external variables, recall decay, and uncontrolled experiential factors that could otherwise affect participant responses between evaluations.

Longer-term follow-up assessments were not included because the primary objective of the study was to capture immediate structural changes in perceived sensory evaluation and PDO-related knowledge rather than long-term retention. Future studies may incorporate delayed post-intervention evaluations to examine persistence and consolidation of acquired knowledge over time.

### 2.6. Educational Intervention

The educational intervention consisted of a standardized face-to-face training session delivered by Dr. María del Rosario Fresno Baquero, Scientific Programmes Coordinator at the Canary Institute for Agrarian Research (ICIA), a senior researcher with extensive expertise in dairy science, goat cheese sensory evaluation, and consumer and expert-panel training methodologies. The instructional component lasted 53 min and 48 s and was delivered uniformly to all participant groups to ensure methodological consistency.

The session content was structured in accordance with the conceptual domains of the questionnaire. The session followed a lecture-based format supported by audiovisual materials, combining structured oral explanations with visual media to facilitate comprehension and maintain consistency across participants/respondents. It began with an introduction to the cultural, productive, and gastronomic relevance of traditional Canarian cheeses, followed by an explanation of the principal sensory dimensions involved in cheese evaluation, including visual, textural, olfactory, gustatory, and temporal attributes. The final section focused on European quality-certification systems, particularly Protected Designation of Origin (PDO) schemes, with detailed explanation of the regulatory requirements and distinguishing characteristics associated with PDO Majorero and PDO Palmero cheeses.

Instruction combined lecture-based explanations, visual support materials, and interactive discussion. In addition to the formal presentation, participants engaged in question-and-answer exchanges lasting approximately 10 ± 2 min in total, allowing clarification of concepts and reinforcement of key information while preserving standardization across sessions.

### 2.7. Statistical Analysis

Because the statistical methodology involved multiple complementary analytical procedures, a summary of the statistical workflow is presented in Table 1. The table outlines each statistical technique, its objective, the principal indicators used, and the criteria applied for interpretation, thereby facilitating comprehension of the analytical strategy and subsequent results.

#### 2.7.1. A Priori Assumptions

Canonical Discriminant Analysis (CDA) requires several assumptions [11,25]. Normality of continuous predictors was checked with the Shapiro–Francia W′ test in Stata 16.0; slight deviations were observed (Supplementary Material S1), but Q–Q plots indicated approximate normality (*p* > 0.05). Categorical variables converted to binary dummies are exempt, and minor deviations are tolerable with adequate sample size [28]. Homogeneity of variance–covariance matrices showed heteroscedasticity via Levene’s test (*p* < 0.05), but CDA is robust for exploratory analyses [41]. Outliers were screened using the ROUT method (Q = 1%) in GraphPad Prism 9.0, with none detected [29,30]. Sample size exceeded ten observations per predictor [31], and VIFs < 5 indicated acceptable multicollinearity [36].

#### 2.7.2. Canonical Discriminant Analysis (CDA)

Canonical Discriminant Analysis (CDA) is a multivariate technique that maximizes separation among predefined groups by constructing linear combinations of variables (canonical functions). It reduces dimensionality and provides clear visualizations of group differences—particularly valuable in studies with multiple responses, factorial structures, or complex interactions. However, classical CDA assumes independence of observations, homogeneity of covariance matrices, and multivariate normality—assumptions violated in repeated measures, where multiple observations per subject introduce within-subject correlations. Ignoring these correlations inflates Type I error, biases estimates, and overstates classification accuracy [41,42,43].

Treating repeated measures as independent discards within-subject dependency, underestimates variances, and misses modeling subject-specific change—the main focus of longitudinal studies. Methods accounting for structured covariance (covariance pattern or mixed-effects models) better handle correlations, unbalanced data, and missing values, yielding more reliable discriminant functions.

Several adaptations of CDA to repeated measures exist: (1) computing change scores (e.g., post–pre differences); (2) aggregating data via means or slopes; or (3) applying multivariate or mixed-effects models that explicitly model within-subject covariance. Among these, graphical CDA provides a robust and intuitive solution [38].

Two graphical outputs are particularly informative:

1. Hypothesis–Error (HE) plots visualize explained (H) vs. residual (E) variation. Ellipses representing group means indicate separation: an elongated H ellipse relative to a circular E ellipse denotes strong discrimination. Wilks’ Lambda is computed as Λ = |E|/|E + H|, with smaller Λ indicating stronger group separation. Permutation tests (e.g., 10,000 iterations) assess significance when assumptions are unmet. Although XLSTAT does not produce HE plots directly, canonical score plots serve as a useful proxy for visualizing group separation.
2. Canonical structure plots show variable contributions as vectors in canonical space—vector length reflects contribution strength, and inter-vector angles indicate correlations [39,40].

By combining CDA with graphical outputs, researchers preserve the dimensionality reduction and interpretive power of CDA while accounting for repeated measures. This approach enables intuitive visualization of group separation, identifies discriminating variables, and respects within-subject correlation, making graphical CDA a powerful method for multivariate repeated-measures analysis.

#### 2.7.3. Explanatory Variables

All survey items served as explanatory variables. These included questions related to general information, self-reported knowledge levels, ability to identify sensory attributes (visual, textural, olfactory, and taste), and knowledge of PDO regulations for Majorero and Palmero cheeses, as well as labeling preferences (see Supplementary Material S1).

#### 2.7.4. Clustering Variable

The grouping variable was the evaluation moment, distinguishing whether the responses were collected before or after the training intervention.

#### 2.7.5. Variable Selection

Variable selection was performed using regularized forward stepwise multinomial logistic regression (SPSS v26.0, IBM, Chicago, IL, USA). Priors were adjusted according to group sizes. Forward and backward approaches yielded the same variables; forward selection was retained for computational efficiency.

#### 2.7.6. Sample Size

Following guidelines recommending at least 20 observations per 4–5 predictors [33], the study exceeded the minimum ratio, ensuring robust CDA results.

#### 2.7.7. Multicollinearity

Multicollinearity, defined as strong intercorrelation among predictors, was assessed using VIF and tolerance. VIF was calculated as:

VIF = 1/(1 − R^2^)

where R^2^ is the coefficient of determination of the regression of each predictor on all others. A threshold of 5 was adopted.

#### 2.7.8. Canonical Correlation and Model Reliability

The number of canonical correlations equals the number of variables in the smaller set. Correlations of 0.30 or greater (roughly 10% explained variance) were considered meaningful.

#### 2.7.9. Tests of Model Adequacy

Bartlett’s Test confirmed adequate correlations (*p* < 0.05), Wilks’ Lambda assessed discriminating power with significance tested via 10,000-iteration permutation in XLSTAT, and Pillai’s Trace was used for robustness to unequal group sizes and assumption violations.

#### 2.7.10. Standardized Discriminant Coefficients and Loadings

Standardized coefficients were used to build the discriminant equations, as these provide a scale-free measure of the relative contribution of each variable to the discriminant function. This is the plain base formula for a discriminant function:

F1 = b1*X1 + b2*X2 + b3*X3 + … + bn*Xn

where

F1 = discriminant score for Function 1;

bi = standardized discriminant coefficient for predictor i;

Xi = standardized value of predictor i.

Standardization was necessary because the predictors were measured on different scales, and unstandardized coefficients would have been dominated by variables with larger variances, making comparisons of their importance misleading.

In contrast, factor loadings (structure coefficients) reflect the correlations between each variable and the discriminant function. To improve interpretability, only variables with loadings greater than or equal to |0.40| were considered meaningful for interpretation. This criterion is widely adopted as it indicates that the variable shares at least a moderate relationship with the discriminant axis, while weaker loadings may reflect noise or incidental associations.

Following the estimation of discriminant functions, group centroids were derived, and squared Mahalanobis distances were computed to quantify separation between groups. The squared Mahalanobis distance measures the distance between group means in multivariate space, while accounting for the variance–covariance structure of the predictors. Larger distances correspond to greater group distinctiveness and stronger discriminant validity of the retained predictors.

Squared Mahalanobis distances were computed as follows to depict the degree of proximity between clusters (before and after training):

D^2^ij = (Yi − Yj) × COV^−1^ × (Yi − Yj)

where D^2^ij is the squared Mahalanobis distance between groups i and j, Yi and Yj are the vectors of group means, and COV^−1^ is the inverse of the covariance matrix.

To facilitate visualization of the relationships among groups, Mahalanobis distances were transformed into Euclidean distances, and hierarchical clustering was performed using the Unweighted Pair Group Method with Arithmetic Mean (UPGMA) implemented in the DendroUPGMA (v 1.0) application [44]. UPGMA is an agglomerative hierarchical clustering algorithm that successively merges the two clusters with the smallest average pairwise distance, recalculating inter-cluster distances as the arithmetic mean of all pairwise distances between observations in the newly formed cluster and the remaining clusters.

This procedure is methodologically justified because Mahalanobis distance accounts for the covariance structure of the data by rescaling and decorrelating the variables, producing an orthogonal standardized space in which Euclidean distances appropriately represent multivariate relationships. Consequently, Euclidean distances are calculated from the transformed canonical space rather than directly from the original ordinal Likert-scale responses. This approach is widely adopted in discriminant, clustering, and ordination analyses to visualize group structure rather than measurement-level differences. The resulting dendrogram therefore reflects the geometrical relationships among the canonical group centroids while maintaining consistency with the discriminant structure obtained from the Canonical Discriminant Analysis. Alternative metrics (e.g., chi-square or corrected Euclidean distances) are more appropriate for frequency or count data; however, after transformation into canonical space, Euclidean distances provide an interpretable and statistically coherent basis for hierarchical clustering.

#### 2.7.11. Cross-Validation

To evaluate the robustness of the discriminant function, classification accuracy was assessed using leave-one-out cross-validation (LOOCV). This approach iteratively omits one observation at a time, builds the discriminant model on the remaining data, and then classifies the omitted case, ensuring that every individual is tested as an independent case. Classification performance was further validated by calculating Press’s Q statistic, which tests whether the observed accuracy exceeds chance classification by at least 25%. The formula applied was:

Q = [(n − (n′ × K))^2^]/[n × (K − 1)]

where n is the total number of observations, n′ is the number of correctly classified cases, and K is the number of groups. Additionally, the discriminant scores were subjected to Receiver Operating Characteristic (ROC) analysis, and the Area Under the Curve (AUC) was calculated as a complementary measure of classification quality, with values closer to 1.0 indicating excellent discrimination.

#### 2.7.12. Post Hoc Statistical Power Analysis

To evaluate the sensitivity of the statistical analyses, a post hoc power analysis was conducted in R (version 4.4.3; R Foundation for Statistical Computing, Vienna, Austria) using RStudio (version 2024.12.1; Posit Software, PBC, Boston, MA, USA). Statistical power (1 − β) was estimated from the non-central F distribution using the observed F statistics derived from the Canonical Discriminant Analysis (Code in Supplementary Material S2), assuming a significance level of α = 0.05 and numerator and denominator degrees of freedom corresponding to the discriminant tests (df_1_ = 1; df_2_ = 528). Power estimates were calculated for the representative F statistics associated with each conceptual domain evaluated in the questionnaire, providing an assessment of the sensitivity of the multivariate comparisons across the different knowledge domains.

## 3. Results

### 3.1. A Priori Assumptions

Preliminary analyses indicated minor deviations from normality in survey responses. The Shapiro–Francia W’ test rejected normality, likely due to non-significant outliers, while Q–Q plots suggested approximate normality (*p* > 0.05). To ensure robustness, all assumptions were verified. Levene’s test showed significant heteroscedasticity (*p* < 0.05), justifying the use of permutation-based canonical correlation analysis, suitable for data violating normality and variance homogeneity. ROUT outlier detection (Q = 1%) in GraphPad Prism 9.0 found no significant outliers.

### 3.2. Descriptive Statistics

Supplementary Table S1 summarizes 112 survey items, including categorical and continuous variables. Categorical items report frequencies and percentages (e.g., Yes/No, True/False, Likert scales), while continuous variables include minimum, maximum, and mean ± SD.

Analysis of categorical items (Questions 1–112) shows strong clustering for many responses; e.g., Question 1 (“Yes” = 99.06%) indicates high consensus. Items with multiple options or “No Opinion” (Questions 37–112) displayed greater variability, reflecting diverse opinions or uncertainty. Continuous variables (Questions 7–36) showed moderate variability (mean ≈ 2.05–3.94 on a 1–5 scale), suggesting generally moderate to high responses with notable individual differences (e.g., Question 13, SD = 1.34).

Question 5 asked about prior sensory analysis course attendance: 39.25% responded “Yes,” 60.75% “No.” Question 6 explored product-specific training among those who answered “Yes,” covering Cheese, Wine, Oil, Beer, Chocolate, Dairy, Honey, Fish, Meat, Coffee, Ham, Whisky, Water, Sparkling Wine, and Milk. Results indicate:

Most common: Cheese (17.74%), Dairy (18.49%), Wine (11.89%).

Less common: Oil (4.34%), Chocolate (1.51%), Coffee (1.13%), Ham (0.75%).

Minimal exposure: Beer, Whisky, Water, Sparkling Wine (<1%).

These findings show sensory analysis training among respondents focuses on core products like cheese, dairy, and wine, with limited attention to other categories, reflecting regional consumption patterns, educational priorities, or availability of product-specific courses.

### 3.3. Canonical Discriminant Analysis

#### 3.3.1. Multicollinearity Analysis

As shown in Supplementary Table S2, multicollinearity was assessed before discriminant analysis using VIF and tolerance values. Across all survey variables (Supplementary Material S1), VIF ranged 1.10–6.34 and tolerance 0.15–0.91.

Within sensory attributes, texture-related variables—external appearance (Q7), cut appearance (Q8), touch texture (Q9), roughness (Q10), elasticity (Q11), humidity (Q12), texture in mouth (Q13), firmness (Q14), friability (Q15), mouth moisture (Q16), adhesiveness (Q17), solubility (Q18), and granularity (Q19)—showed substantial overlap. Humidity (Q12), friability (Q15), and mouth moisture (Q16) exceeded thresholds (VIF > 5, tolerance < 0.20) and were excluded. Elasticity (Q11) and firmness (Q14) were retained due to higher discriminatory weight (VIF ≈ 3.8). Olfactory and taste items all met criteria (VIF < 3.5, tolerance > 0.30) and were retained.

For PDO knowledge (Q34–62), items Q34, Q35, Q41, Q43, Q45, and Q47 exceeded VIF > 5 and were collapsed into a composite “PDO knowledge” score, while regulation-related items (Q38, Q39, Q49, Q52, Q55, Q60, Q62) were retained individually. Similar consolidation was applied to PDO Majorero (Q63–82) and PDO Palmero (Q83–102) blocks, collapsing near-identical items (e.g., Q63 vs. Q64; Q83 vs. Q84) to avoid redundancy.

Labeling preferences (Q103–109) showed moderate multicollinearity (VIF 2.1–3.9, tolerance > 0.25), and all five priority items were retained. After these refinements, the final variable set had VIF < 3.0 and tolerance > 0.35, meeting criteria for inclusion in the discriminant analysis.

#### 3.3.2. Canonical Correlation Dimensions, Efficiency and Model Reliability

##### Canonical Correlation Dimensions and Bartlett’s Test

The discriminant analysis produced a single canonical function (F1) with an eigenvalue of 3.41, explaining 100% of the discriminatory power. This indicates that nearly all variance separating the predefined groups is captured by the first dimension, reflecting strong group separation. Bartlett’s test confirmed the eigenvalue’s significance (χ^2^ = 674.84, *p* < 0.001), supporting the robustness of F1. Although a second dimension could contribute marginally, its explanatory power is negligible, so interpretation reliably relies on the first canonical root.

##### Wilks’ Lambda Test

Analysis of group mean differences before and after training (Supplementary Table S3; summarized conceptually in Table 2) demonstrated a strong overall training effect across most conceptual domains evaluated in the questionnaire. The most pronounced changes were observed in the domains of milk-origin and species restrictions, PDO territorial linkage, and sensory texture perception, particularly in items related to the exclusive use of authorized milk species, the geographical exclusivity of Majorero PDO production, and texture perception in mouth (Wilks’ Lambda = 0.6345–0.7256; F = 199.66–304.20; *p* < 0.0001). As shown in Table 2, these results indicate that the training sessions were especially effective in correcting misconceptions associated with PDO territoriality, milk composition regulations, and the interpretation of sensory texture attributes. Similarly, Supplementary Table S3 revealed substantial reductions in “No Opinion/No Response” selections across numerous PDO-related items, suggesting a marked decrease in participant uncertainty and greater confidence in regulatory interpretation after training.

Significant improvements were also identified in PDO labeling and traceability systems, PDO technological and cheesemaking regulations, and native breed recognition and herdbook registration. Variables associated with numbered counter-label requirements, registered dairies, cheesemaking specifications, coagulation practices, and the use of authorized coagulants generally presented Wilks’ Lambda values between 0.79 and 0.95 and F-values ranging from approximately 20 to 135 (*p* < 0.0001) in Supplementary Table S3, while Table 2 grouped these variables into broader conceptual domains to facilitate interpretation. Likewise, concepts related to the role of native breeds, herdbook registration systems, and breed identity within both Majorero and Palmero PDOs showed strong statistical support, confirming that the training effectively reinforced understanding of the biological and territorial foundations underlying PDO certification systems.

Training effects were also evident in sensory-analysis competencies. According to Supplementary Table S3 and the conceptual synthesis presented in Table 2, significant differences were detected in the identification and interpretation of texture-, olfactory-, and gustatory-related sensory descriptors, including friability, adhesiveness, humidity, aroma intensity, dairy and plant aromatic families, and acidic, bitter, salty, and spicy taste attributes. The strongest effects within the sensory domain were observed in texture-related descriptors (Wilks’ Lambda = 0.7256–0.8891; F = 65.84–199.66; *p* < 0.0001), whereas some olfactory and gustatory variables, such as roasted or spice-related aromatic families and sweet or astringent tastes, showed weaker or non-significant differences after training. These findings suggest that participants improved more consistently in sensory dimensions directly addressed during practical evaluation sessions.

Additional conceptual gains summarized in Table 2 included improvements in PDO governance and management, reductions in hygienic–sanitary and sustainability misconceptions, and better understanding of the economic and regulatory functions of PDO systems. Participants demonstrated greater awareness of the role of producers, administration, and Regulatory Councils within PDO governance structures, as well as improved recognition that PDO systems are not exclusively linked to hygienic guarantees, ecological production, or anti-fraud mechanisms. Improvements were also observed in participants’ self-perceived knowledge of PDOs and sensory analysis, indicating that training positively influenced both objective and subjective dimensions of knowledge acquisition.

In contrast, several variables associated with previous sensory-analysis experience, stable consumption preferences, selected labeling-preference rankings, and some already consolidated PDO concepts showed limited or non-significant variation between evaluation moments in Supplementary Table S3 (Wilks’ Lambda ≈ 1.0; F < 3; *p* > 0.05). As summarized in Table 2, these stable variables likely reflect concepts that participants already understood adequately before training or domains unrelated to the educational intervention itself. Overall, the results presented in Supplementary Table S3 and synthesized in Table 2 confirm that the training sessions substantially strengthened both sensory-analysis competencies and understanding of PDO regulatory frameworks, while simultaneously reducing uncertainty and improving interpretation of complex territorial, technological, and traceability-related concepts.

##### Pillai’s Trace Criterion

The multivariate analysis based on Pillai’s Trace confirmed a strong overall effect. The obtained value of Pillai’s Trace (0.7731) indicates that a substantial proportion of the variance in the dependent variables is explained by the factor under study. The observed F statistic (F(146, 383) = 8.94) was markedly higher than the critical value (1.25), and the associated *p*-value (<0.0001) demonstrated that the effect was highly significant at the 0.05 level. These results lead to the rejection of the null hypothesis of no group differences, supporting the conclusion that the groups differ significantly across the combined set of dependent variables.

##### Canonical Standardized Coefficients, Loadings, and Spatial Representation

**Figure 3:**
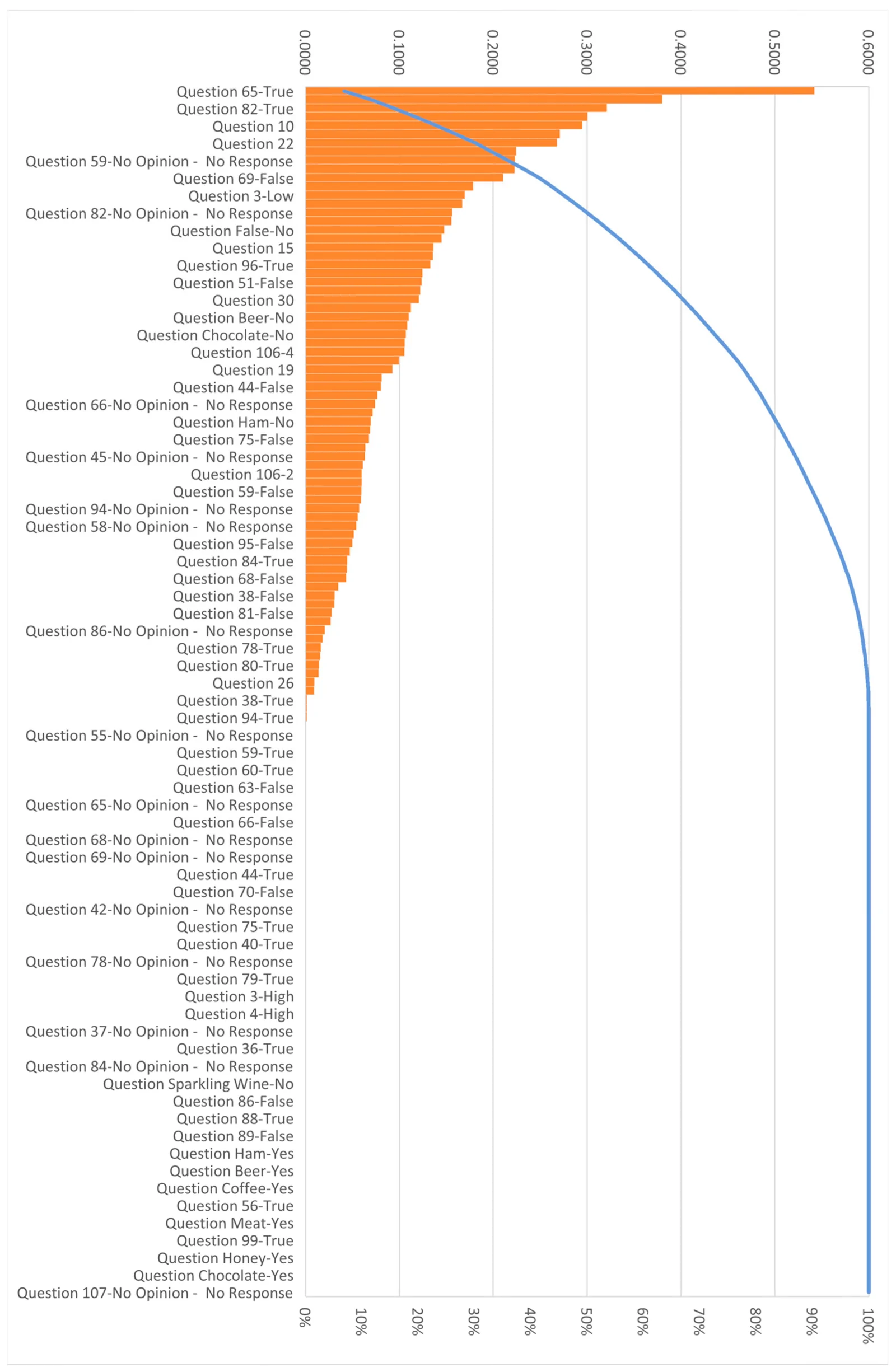
Pareto chart of the standardized discriminant coefficients for the questionnaire variables retained in the Canonical Discriminant Analysis. Orange bars represent the standardized discriminant coefficients, indicating the relative contribution of each retained variable to the discriminant function, ordered from highest to lowest. The blue line shows the cumulative contribution of the retained variables to the overall discriminatory capacity of the model.

Discriminant Function 1 (F1) was constructed as a linear combination of all variables weighted by their standardized discriminant coefficients (Figure 3 and Supplementary Material S3). Large positive contributors—Q65_True (0.5421), Q82_True (0.3210), Q13 (0.3005), Q10 (0.2951), Q22 (0.2679)—indicate that strong affirmative responses increase the discriminant score, positioning respondents toward the positive pole. Key negative contributors—Q73_NoOpinion (−0.3680), Q31 (−0.3413), Q78_False (−0.2736), Q9 (−0.2818), Q107_2 (−0.1974)—show that indecision, neutrality, or rejection push individuals toward the opposite pole. F1 thus primarily reflects engagement versus detachment, distinguishing consistent affirmative respondents from hesitant or negative ones. Intermediate coefficients capture subtler attitude differences, emphasizing decisiveness and positive endorsement as central to group differentiation.

In this discriminant analysis, the first function (F1) effectively separates participants with stronger knowledge and training from those with weaker or uncertain profiles. High positive coefficients are linked to correct recognition of PDO regulations—e.g., allowance of up to 15% Canary sheep milk in Majorero (Q65 True), exclusive PDO protection for registered cheeses (Q82 True), and geographical production limits (Q69 False)—and to accurate discrimination of complex sensory traits like roughness, texture in mouth, and bitterness. Strong negative coefficients correspond to misconceptions or indecision, particularly “No Opinion” responses (Q73, Q76, Q101) and difficulties identifying sensory attributes such as aftertaste. F1 thus distinguishes knowledgeable, confident, and sensory-trained respondents from uncertain, less trained, or misinformed ones.

**Figure 4:**
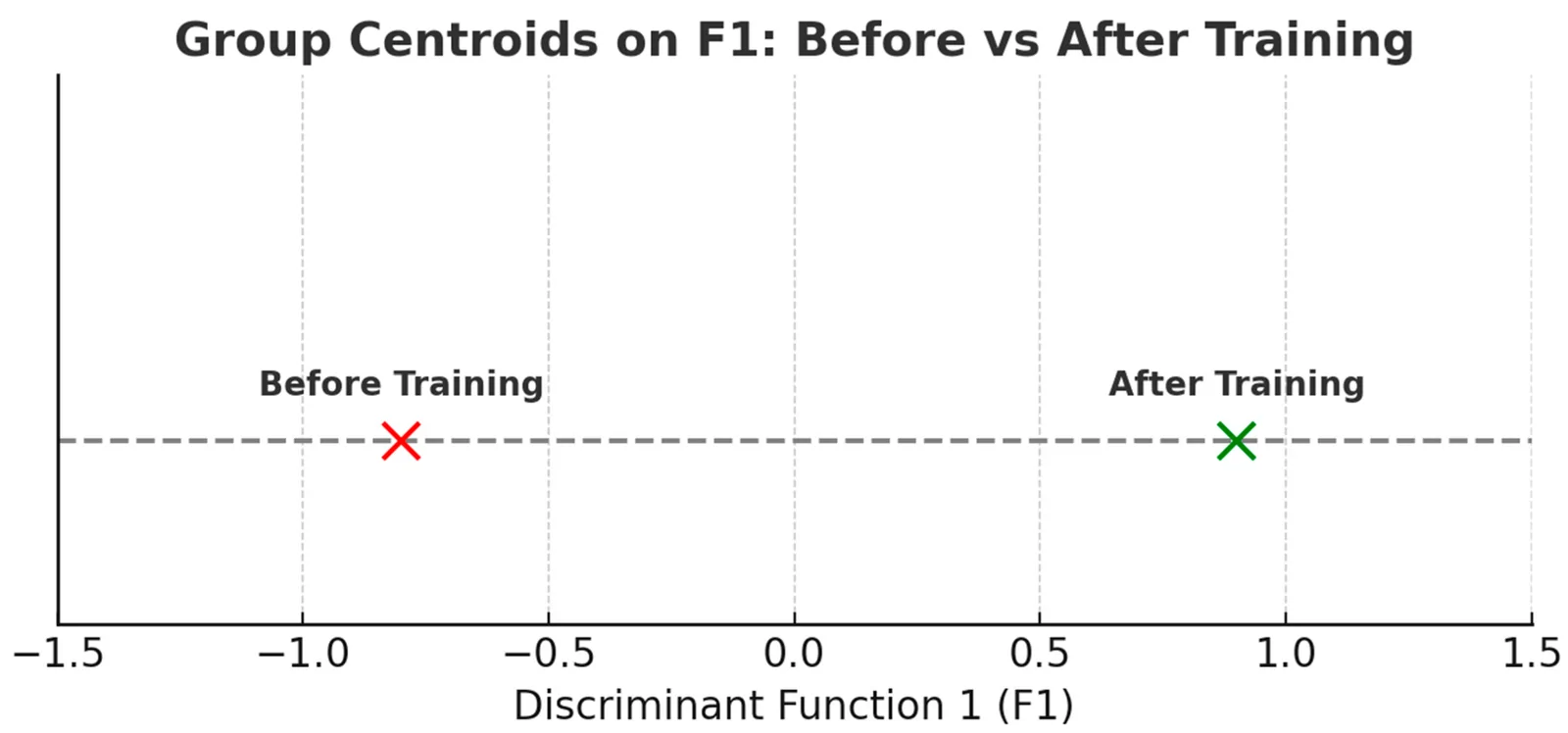
Canonical structure vector plots for discriminant loadings for survey question traits.

Graphical centroids along F1 show clear separation between pre- and post-training participants (Figure 4). Pre-training respondents cluster on the negative side, with more “No Opinion” responses, weaker PDO knowledge, and lower sensory discrimination. Post-training respondents occupy the positive side, reflecting improved regulatory understanding, confidence, and sensory skills.

**Figure 5:**
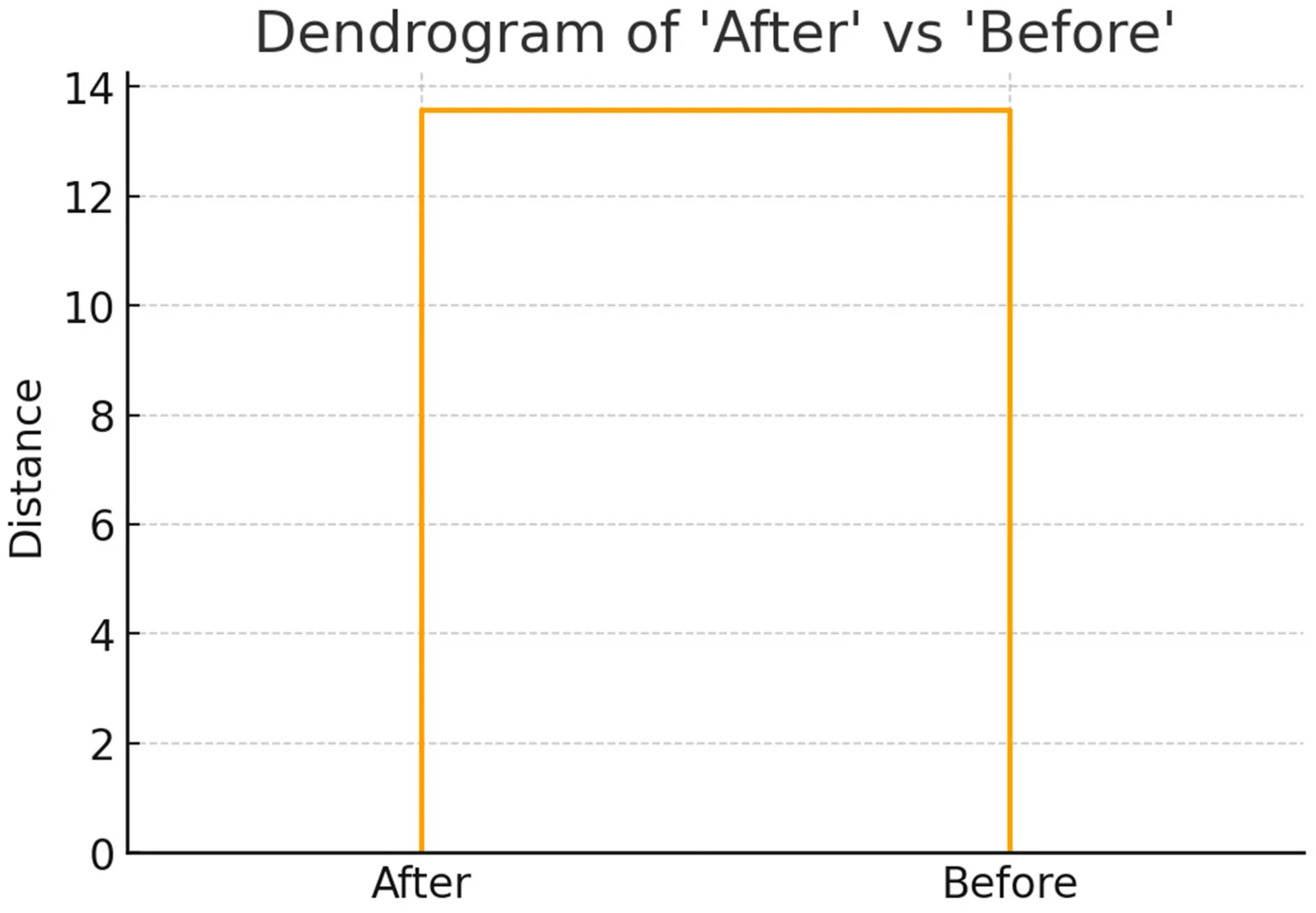
Dendrogram constructed from Mahalanobis’s distances across evaluation moments.

Hierarchical clustering of the two time points (Figure 5) shows a single linkage step of 13.58 units, indicating substantial multivariate separation between groups and visually confirming that training produced a measurable divergence in participant profiles.

##### Cross-Validation

**Figure 6:**
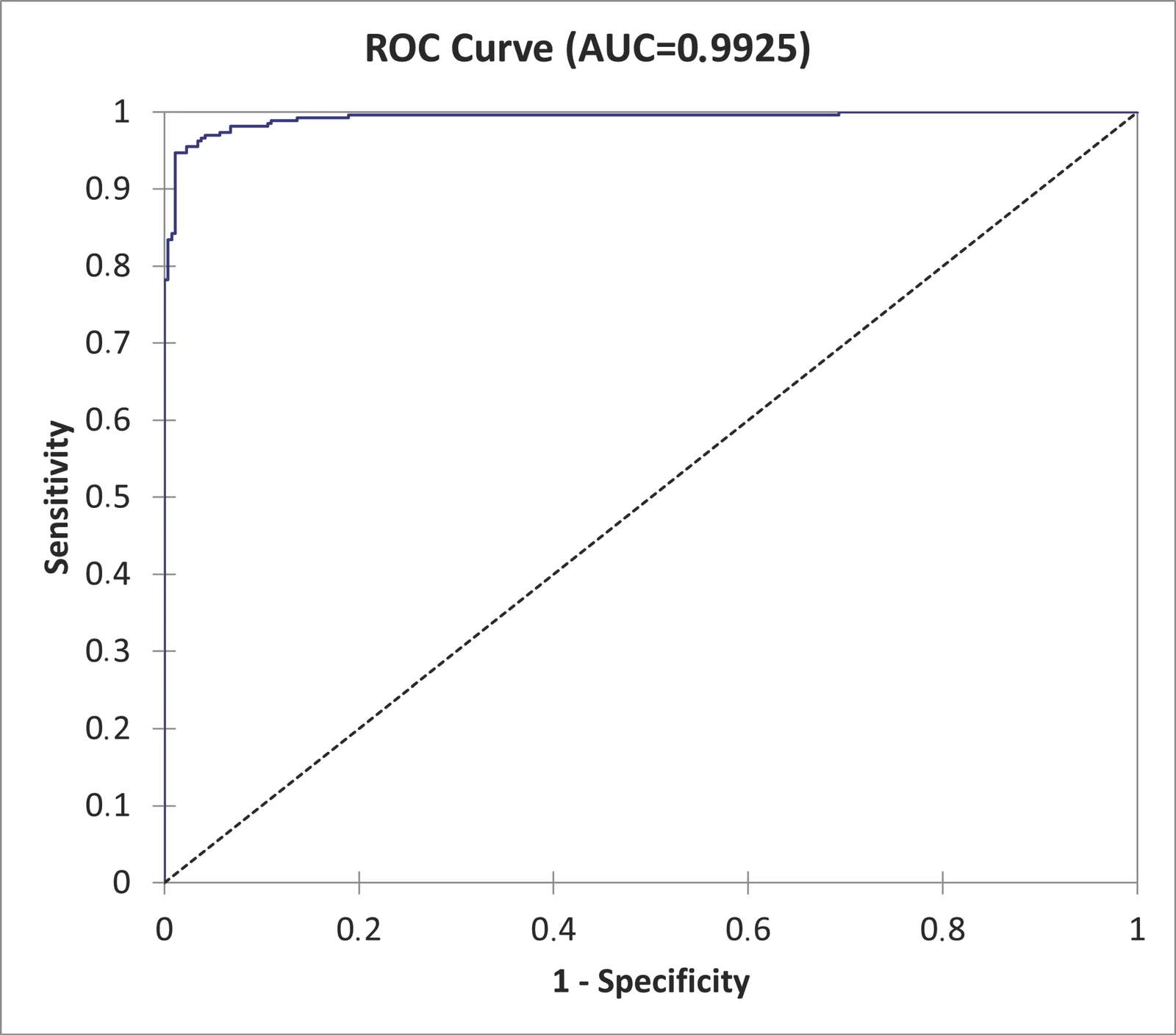
Receiver Operating Characteristic (ROC) curve illustrating the Area Under the Curve (AUC) for discriminant analysis, reflecting the model’s ability to distinguish between evaluation timing classes.

The discriminant analysis correctly classified 96.23% of cases under LOOCV: 96.59% for the “After training” group and 95.86% for “Before training,” with only 20 misclassifications out of 530. Press’s Q confirmed accuracy was significantly above chance (*p* < 0.001). The ROC curve showed excellent discriminatory capacity, with an AUC of 0.9925 (Figure 6), corroborating that the discriminant function effectively separates pre- and post-training participants.

### 3.4. Post Hoc Statistical Power Analysis

The post hoc power analysis indicated that the discriminant analyses were generally associated with high statistical sensitivity. Most conceptual domains yielded observed power values exceeding 0.99, reflecting the large sample size (*n* = 530) and the substantial effect sizes detected by the Canonical Discriminant Analysis. Lower power values were observed only for domains with weak or non-significant effects, such as *Labeling Preferences* (power = 0.780) and *Stable Variables* (power = 0.371), consistent with their small observed F statistics and limited discriminative ability between pre- and post-training evaluations. Overall, the mean observed statistical power across all conceptual domains was 0.947, indicating that the study sample provided adequate statistical sensitivity to detect meaningful multivariate differences for the vast majority of the evaluated domains.

## 4. Discussion

Although previous literature has extensively examined consumer attitudes toward PDO foods, authenticity, and purchasing behavior, comparatively limited attention has been devoted to the methodological validation of consumer-perception surveys themselves. Existing studies have frequently prioritized descriptive market outcomes without rigorously evaluating whether survey instruments possess sufficient discriminatory power, classification robustness, and predictive stability across heterogeneous consumer groups.

The present study addresses this gap by proposing a methodological framework that combines CDA with cross-validation procedures for the validation and optimization of consumer-perception surveys applied to traditional PDO cheeses. Specifically, the study evaluates the discriminative capacity of questionnaire items related to PDO knowledge, trust, authenticity, sensory perception, and purchasing behavior, while simultaneously assessing model robustness and predictive stability through LOOCV and K-fold validation procedures. By integrating multivariate discrimination with robust validation techniques, the study contributes to the methodological advancement of PDO-food consumer research and provides a replicable analytical framework applicable to other traditional foods derived from autochthonous breeds.

Respondents’ answers highlighted concerns about the authenticity of Majorero and Palmero PDO cheeses, emphasizing the need for accurate labeling. Official labels not only convey origin and quality but also build trust and prevent fraud [16]. Similarly, origin labeling increases perceived value and motivates consumers to pay a premium, serving as a reliable indicator of authenticity [22]. Information on origin and animal breed further strengthens perceived quality by linking the product to its production environment [45]. Consumers also focus on authenticity, origin, and quality attributes on labels, which strongly influence purchasing decisions, as reflected in this study [46].

Respondents’ opinions were divided regarding the relevance of breed in PDO cheeses. Some considered it a key factor for ensuring authenticity, while others prioritized origin certification over breed. This perception aligns with Menozzi et al. [20], who observed that for cheeses such as Parmigiano Reggiano and Comté, consumers valued the geographical indication and certified quality more than the explicit mention of animal breed.

The results revealed a direct relationship between territorial attachment and knowledge of Canarian PDO cheeses (Palmero and Majorero) among island residents. Inhabitants of La Palma and Fuerteventura demonstrated greater familiarity with these products than those from Gran Canaria. Teuber [21] compared familiarity and knowledge regarding Protected Geographical Indications (PGIs) and conducted a survey of local consumers, who showed higher awareness than non-local consumers. Recent studies further suggest that younger consumers, particularly Generation Z, show increasing interest in local gastronomy, traditional foods, and rural authenticity, which may strengthen engagement with region-linked PDO products [47]. Likewise, research comparing local and non-local consumers indicates that residents tend to prioritize regional origin more strongly than PDO certification itself, whereas consumers farther from the production area often place greater value on the PDO seal as an indicator of authenticity and quality [48]. However, evidence directly comparing younger and older populations regarding territorial attachment and PDO cheese knowledge remains limited. Therefore, the demographic composition of the present sample should be considered when interpreting these findings, since approximately 75% of respondents were under 52 years of age. Younger consumers may express territorial attachment differently from older populations, whose perceptions are often shaped by stronger intergenerational traditions, long-term consumption habits, and deeper cultural ties to local products. Consequently, the observed relationship between territorial attachment and PDO cheese knowledge may not be fully generalizable to older consumer groups, and future studies should include more balanced age distributions to better evaluate potential generational differences.

The findings indicated that consumers’ perception of milk composition and origin in Majorero and Palmero PDO cheeses relied more on trust in certification than on detailed technical knowledge. As Bouhaddane et al. [49] noted, the legitimacy of PDO labeling was primarily interpreted through symbols and subjective perceptions. In this context, it is relevant that Majorero cheese regulations strictly specified the permitted raw material: exclusively Majorera goat milk, allowing only up to 15% Canarian sheep milk in cheeses intended for maturation [8]. However, most consumers were unaware of this regulation, indicating that authenticity was valued more for its association with the PDO seal and local tradition than for actual knowledge of composition.

Perception of PDO products depended more on consumer experience and familiarity than on regulatory knowledge. Trust in the seal fostered satisfaction and loyalty primarily among prior consumers [22]. Studies on Idiazabal cheese showed consumer acceptance often diverged from technical quality assessments, reflecting perception’s subjective nature [50]. Similarly, PDO labeling did not always boost purchase intention, as some consumers viewed it as added value or marketing rather than a regulatory guarantee [27]. This confusion may stem from limited awareness that PDO oversight lies mainly with independent Regulatory Councils, not public administration, sometimes causing misinterpretation even among industry professionals.

In this study, the question regarding the paprika coating of PDO cheeses did not measure technical knowledge but rather the respondents’ direct experience. Various studies have shown that responses related to visible attributes were mainly based on familiarity and subjective consumer knowledge rather than objective regulatory knowledge [51]. Specific cheese studies demonstrated that regional familiarity and expectations influenced sensory acceptance and interpretation of visible attributes [52], and that visible elements, such as paprika or rind color, affected choice and perception of intensity among different consumers [53].

The survey statement on the influence of goat feeding on Palmero and Majorero PDO cheeses likely did not assess specific technical knowledge but rather respondents’ intuition. This aligns with recent findings showing that, regarding feeding-related questions, the population generally has limited knowledge and responds based on intuition or beliefs rather than technical information [46].

Clarkson et al. [54] noted that liking a product motivates consumers to gain more knowledge, either by expanding experience if novice or deepening it if expert. This explains why respondents in our study showed greater knowledge of Majorero and Palmero PDO cheeses. Preference for these cheeses reflected both taste and familiarity, enhancing their ability to recognize, evaluate, and differentiate products. Greater category knowledge also improves interpretation of extrinsic cues—price, brand, labeling—supporting informed decisions. Our findings on the need for specific PDO cheese training align with prior research, showing it effectively increases consumer knowledge, quality evaluation skills, and market differentiation [55].

Finally, surveyed consumers associated the PDO seal with greater food safety. According to Bravo et al. [24], certification was not an attribute of food safety but an indicator of product quality. The study showed that consumer perception of PDO products was one of quality and, therefore, of greater food safety. Thus, the idea that certification equated to safety represented a misconception, reflecting a significant knowledge gap regarding quality seals. Previous studies have consistently documented confusion and misinterpretation regarding PDO and PGI labels, with consumers frequently misunderstanding the scope and guarantees associated with these certifications [56]. Information asymmetry and limited familiarity with regulatory frameworks may lead consumers to overgeneralize the meaning of quality labels, associating them not only with authenticity and origin but also with health and food safety assurances [57]. Some studies have also identified health and safety perceptions as important drivers of consumer trust in geographically indicated products among younger populations [58]. However, the available literature does not provide representative prevalence estimates of how many consumers explicitly believe that PDO certification guarantees sanitary safety, indicating that this misconception remains insufficiently quantified in the broader population.

The persistence of this misconception even after training suggests that certain beliefs may be strongly rooted in heuristic associations between certification, quality, and safety. Consumers often interpret official seals as comprehensive guarantees covering all aspects of the product, including sanitary control, despite PDO schemes being primarily focused on origin, production methods, and product authenticity. This interpretation is consistent with previous research showing that short educational interventions can improve factual food-related knowledge while remaining less effective at modifying emotionally grounded or intuitive beliefs [59]. Studies on food hygiene education have demonstrated significant short-term gains in objective knowledge after training interventions [58], whereas research on belief perseverance indicates that emotionally or identity-linked beliefs frequently resist factual correction [59]. Therefore, future educational strategies may benefit from explicitly distinguishing between quality certification and food safety regulation while incorporating more interactive or experience-based learning approaches capable of addressing deeply rooted intuitive associations more effectively.

Canonical Discriminant Analysis (CDA) was selected because it enabled the identification of multivariate patterns that differentiated consumer perceptions before and after training while also providing an interpretable low-dimensional representation of group separation. CDA has been widely applied in behavioral and consumer research for exploratory classification purposes and is particularly useful when the objective is to examine how multiple perception variables jointly contribute to group differentiation [32,33,34]. Although some assumptions of CDA may be moderately violated in practice, previous studies have shown the method to remain robust when sample sizes are adequate and the analysis is exploratory in nature [10]. Therefore, CDA was considered an appropriate tool for evaluating perceptual shifts associated with the training intervention.

From a practical perspective, these findings indicate that educational interventions influence consumer perceptions primarily by strengthening familiarity with authenticity cues, territorial identity, and confidence in PDO certification rather than by increasing technical knowledge alone. This suggests that educational and promotional initiatives should prioritize experiential learning and sensory training to improve consumer understanding and appreciation of traditional cheeses.

Taken together, these findings reinforce that the main contribution of the present study lies in improving understanding of how familiarity, trust in certification, territorial attachment, and prior sensory experience shape consumer perceptions of PDO cheeses, as well as how targeted training interventions may enhance consumer knowledge and interpretation of quality labels.

From a practical perspective, these findings indicate that educational interventions improve consumer perceptions of PDO cheeses primarily by reinforcing familiarity with authenticity cues, sensory characteristics, territorial identity, and confidence in PDO certification rather than by increasing technical knowledge alone. This suggests that communication and educational strategies should prioritize experiential learning and sensory training, as these dimensions appear to drive consumer confidence, product appreciation, and more informed purchasing decisions more effectively than detailed regulatory information alone.

Although logistic regression is widely used for modeling categorical outcomes, Canonical Discriminant Analysis (CDA) is particularly appropriate when the objective is to maximize discrimination among predefined groups. CDA identifies linear combinations of predictors that maximize group separation, reduces data dimensionality through canonical functions, and facilitates interpretation of complex multivariate relationships. It is also robust to complete or near-complete separation and to multicollinearity, making it especially suitable for behavioral and consumer research involving multiple correlated predictors [20,53,54].

Although CDA assumes multivariate normality and homogeneity of covariance matrices, moderate violations are generally tolerated in exploratory studies with adequate sample sizes [10]. Accordingly, despite the heteroscedasticity detected by Levene’s test (*p* < 0.05), CDA was considered appropriate for identifying group-level perceptual differences and examining changes in consumer perceptions before and after the educational intervention.

The present study has several limitations that should be considered when interpreting these findings. First, participants were recruited using a non-probabilistic convenience sampling strategy during organized educational activities, which may limit the generalizability of the results to the wider population of cheese consumers. Although information on age, gender, profession, institutional affiliation, and island of recruitment was collected, other socio-demographic variables that may influence consumer perceptions of PDO products, including educational attainment, household income, region of residence, and urban–rural residency, were not assessed. The absence of these variables limited our ability to evaluate their potential influence on consumer knowledge, attitudes, trust, and purchasing behaviour. Second, the questionnaire was administered immediately before and after the educational intervention, allowing assessment of short-term learning but not the persistence of knowledge acquisition or changes in purchasing behaviour over time. Third, although Majorero and Palmero PDO cheeses provide an excellent model for studying certified traditional foods, consumer perceptions may differ across other PDO products, cultural settings, and certification schemes. Future studies should therefore include probabilistic sampling strategies, a broader range of socio-demographic variables, long-term follow-up assessments, and external validation in additional PDO food systems. Furthermore, although specific measures were implemented to minimize information exchange between successive training groups (independent sessions, standardized protocol, and instructions not to share questionnaire content), the possibility of informal communication between participants cannot be completely excluded. Consequently, a small degree of information contamination cannot be entirely ruled out, although any potential impact is expected to be minimal.

## 5. Conclusions

This study demonstrates that consumer perception of PDO cheeses is influenced primarily by familiarity, trust in certification systems, territorial identity, and sensory experience rather than by detailed technical knowledge of PDO regulations. Educational interventions improved consumer understanding, reduced misconceptions, and reinforced recognition of authenticity cues, highlighting the value of training for strengthening food-label literacy and appreciation of traditional dairy products. From a methodological perspective, the study shows that Canonical Discriminant Analysis (CDA), combined with cross-validation procedures, provides a robust framework for validating consumer-perception surveys by simultaneously evaluating discriminatory capacity, classification performance, and predictive stability. The identified discriminant variables also provide a basis for developing shorter and more practical survey instruments. Several limitations should be acknowledged. The study focused exclusively on two PDO cheeses from the Canary Islands, limiting the generalizability of the findings to other products or cultural contexts. In addition, the immediate pre–post evaluation design assessed short-term learning effects rather than long-term knowledge retention, and the 112-item questionnaire may increase respondent burden. Future research should validate the proposed framework in other PDO and PGI food systems, evaluate the persistence of educational effects through longitudinal designs, and develop optimized survey versions based on the most informative discriminant variables. Overall, these findings demonstrate that educational interventions can enhance consumer understanding of PDO certification and support informed purchasing decisions, contributing to the sustainable promotion of traditional products linked to autochthonous breeds.

## Figures and Tables

**Table 1:** Summary of the statistical techniques applied, their corresponding indicators, and interpretation criteria used throughout the study.

Statistical Technique	Purpose	Main Indicators/Statistics	Interpretation	References
Assumption checking	Verify suitability of the data for multivariate analyses	Shapiro–Francia test, Q–Q plots, Levene’s test, VIF, ROUT outlier detection	Normality, homogeneity, absence of influential outliers, and acceptable multicollinearity (VIF < 5) support the validity of subsequent analyses.	[25,11,26,27,28,29,30,31,32,33,34,35,36]
Canonical Discriminant Analysis (CDA)	Identify variables that best discriminate between pre- and post-training responses	Canonical functions, canonical correlations, standardized discriminant coefficients, structure coefficients (loadings)	Larger canonical correlations and higher absolute loadings indicate variables with greater discriminatory capacity.	[38,39,40,8,41,42,43]
Variable selection	Select the most informative predictors for discrimination	Forward stepwise multinomial logistic regression	Retains variables providing the greatest discriminatory information while reducing redundancy.	[38,39,40,8,41,42,43]
Model adequacy	Evaluate the statistical significance and robustness of the discriminant model	Wilks’ Lambda, Bartlett’s test, Pillai’s Trace	Smaller Wilks’ Lambda and significant Bartlett’s test indicate better group discrimination; higher Pillai’s Trace reflects stronger multivariate effects and robustness.	[38,39,40,8,41,42,43]
Group separation	Quantify discrimination between groups	Group centroids, squared Mahalanobis distances	Greater distances indicate stronger separation between pre- and post-training groups.	[44]
Hierarchical clustering	Visualize relationships among groups	Euclidean distances derived from Mahalanobis distances; dendrogram	Closer clusters indicate greater similarity; larger branch lengths indicate greater dissimilarity.	[44]
Cross-validation	Assess predictive robustness and generalizability	Leave-One-Out Cross-Validation (LOOCV), classification accuracy	Higher classification accuracy indicates greater predictive performance and model stability.	[2,3,25]
Classification validation	Determine whether classification exceeds chance expectations	Press’s Q statistic	Significant Press’s Q indicates that classification accuracy is significantly better than chance.	[2,3,25]
Diagnostic performance	Evaluate the model’s ability to distinguish between groups	ROC curve, Area Under the Curve (AUC)	AUC values close to 1.0 indicate excellent discriminatory performance; values around 0.5 indicate no discrimination.	[2,3,25]

**Table 2:** Summary of the main conceptual domains evaluated in the questionnaire, including representative items, principal PDO- and sensory-related topics, statistical support (Wilks’ Lambda and F-values), significance levels, and the general changes observed between pre- and post-training evaluations. This table synthesizes the extended results presented in Supplementary Table S3.

Main Conceptual Domain	Representative Questionnaire Items	Principal Topics Evaluated	Statistical Support (Wilks’ Lambda/F-value)	Significance Level	General Outcome After Training
Self-perceived knowledge and prior experience	Q3, Q4, Q5	Knowledge of PDOs, sensory analysis, and previous training experience	0.8394–0.9060/54.77–101.00	*p* < 0.0001	Participants reported higher levels of perceived knowledge in PDOs and sensory analysis, while previous training experience variables remained largely stable
Sensory texture perception	Q9–19	Texture in mouth, friability, moisture, firmness, adhesiveness, granularity, elasticity, roughness, solubility	0.7256–0.9864/7.26–199.66	*p* < 0.0001 to *p* = 0.0073	Significant improvements were observed in participants’ ability to identify and interpret texture-related sensory descriptors
Olfactory perception	Q20–27	Aroma intensity, dairy, plant, roasted, spice, and additional aromatic families	0.8522–0.9986/0.73–91.60	*p* < 0.0001 to *p* = 0.3946	Training improved recognition of olfactory descriptors, particularly dairy and plant aromatic families
Gustatory perception	Q28–33	Salty, acidic, bitter, spicy, sweet, and astringent tastes	0.8879–0.9999/0.05–66.67	*p* < 0.0001 to *p* = 0.8319	Participants improved recognition of several gustatory descriptors, especially acidic, bitter, salty, and spicy perceptions
PDO sanitary and sustainability misconceptions	Q37–40	Hygienic-sanitary guarantees, ecological production, fraud prevention, added value	0.9241–0.9997/0.18–43.34	*p* < 0.0001 to *p* = 0.6688	Training reduced misconceptions linking PDO certification exclusively with food safety, sustainability, or anti-fraud functions
PDO governance and management	Q41, Q42, Q49	Role of farmers, administration, and PDO management systems	0.9595–0.9995/0.28–22.31	*p* < 0.0001 to *p* = 0.5987	Improved understanding of governance structures and administrative responsibilities within PDO systems
PDO territorial linkage and geographical restrictions	Q44, Q53, Q55–57, Q62, Q71, Q73, Q92	Territorial identity, production areas, maturation restrictions, grazing limitations	0.7238–0.9999/0.03–201.52	*p* < 0.0001 to *p* = 0.8595	Strong improvements were observed in understanding geographical exclusivity and territorial production restrictions
PDO labeling and traceability systems	Q45, Q51, Q75, Q84, Q85, Q94	Numbered counter-labels, registered dairies, traceability requirements	0.7962–0.9998/0.09–135.11	*p* < 0.0001 to *p* = 0.7688	Participants showed greater understanding of official traceability systems and mandatory labeling requirements
PDO technological and cheesemaking regulations	Q58–60, Q78, Q79, Q99	Cheesemaking specifications, coagulation temperatures, coagulants, technological restrictions	0.8075–0.9999/0.03–125.90	*p* < 0.0001 to *p* = 0.8536	Training improved recognition of technological requirements and permitted traditional cheesemaking practices
Milk-origin and species restrictions	Q65, Q66, Q68, Q86	Authorized milk species, breed-specific milk use, sheep milk allowance	0.6345–0.9924/4.04–304.20	*p* < 0.0001 to *p* = 0.0449	Significant reductions in misconceptions regarding milk composition and authorized species were observed
Native breeds and herdbook registration	Q69, Q70, Q80, Q81, Q88, Q89, Q101	Breed standards, herdbook registration, importance of native breeds	0.8220–0.9997/0.16–114.30	*p* < 0.0001 to *p* = 0.6915	Participants demonstrated improved understanding of breed identity and the role of native breeds in PDO certification
Majorero PDO commercialization requirements	Q82, Q84, Q85	Registered dairies and commercialization rules	0.8665–0.9990/0.55–81.33	*p* < 0.0001 to *p* = 0.4601	Increased awareness of the legal requirements necessary to market cheeses under the Majorero PDO
Palmero PDO commercialization requirements	Q94–96	Labeling, counter-labels, coatings, fresh-cheese protection	0.7590–1.0000/0.00–167.68	*p* < 0.0001 to *p* = 0.9713	Improved understanding of Palmero PDO labeling and commercialization regulations
Labeling preferences	Q106, Q107	Importance of island and breed identification on labels	0.9860–1.0000/0.00–7.49	*p* = 0.0064 to *p* = 0.9713	Participants increasingly prioritized breed and territorial identity as differentiating labeling elements
Reduction in uncertainty responses	Multiple NONR items	“No Opinion/No Response” selections across PDO and sensory questions	0.7623–0.9879/6.47–164.62	*p* < 0.0001 to *p* = 0.0113	Training substantially reduced uncertainty and indecision across most conceptual domains
Stable or non-significant variables	Product-experience variables and selected PDO items	Previous product experience, some already consolidated PDO concepts	0.9950–1.0000/0.00–2.67	*p* = 0.1028 to *p* = 0.9957	Several variables remained stable due to already high baseline knowledge or low misconception prevalence

## Data Availability

The original contributions presented in this study are included in the article/Supplementary Material. Further inquiries can be directed to the corresponding author.

## Supplementary Materials

The following supporting information can be downloaded at https://www.mdpi.com/article/10.3390/foods15162881/s1, Material S1: Survey Questionnaire (English Translation); Material S2: Post Hoc Power Analysis for Canonical Discriminant Analysis. R script used to estimate post hoc statistical power (1 − β) from the observed F statistics of the Canonical Discriminant Analysis using the non-central F distribution. The script calculates power for each conceptual domain evaluated in the questionnaire based on the representative F statistics and corresponding degrees of freedom; Material S3: Discriminant Function 1 (F1); Table S1. Descriptive statistics of survey responses. For categorical variables, frequencies and percentages are reported; for continuous variables, minimum, maximum, and mean ± standard deviation are presented; Table S2. Multicollinearity Analysis of Survey Questions; Table S3. Results for the tests of equality of group means to test for difference in the means depending on evaluation moment (before or after training).

## Abbreviations

The following abbreviations are used in this manuscript:

PDO	Protected Designation of Origin
EU	European Union
ICIA	Instituto Canario de Investigaciones Agrarias
ANCAM	Asociación Nacional de Criadores de Cabras de Raza Majorera
ACP	Asociación de Criadores de Cabra Palmera
CDA	Canonical Discriminant Analysis
LOOCV	Leave-One-Out Cross-Validation
ROC	Receiver Operating Characteristic
AUC	Area Under the Curve
VIF	Variance Inflation Factor
ROUT	Robust Regression and Outlier Removal
HE	Hypothesis–Error
PGI	Protected Geographical Indication
SD	Standard Deviation
SPSS	Statistical Package for the Social Sciences
