# Supplementary material for: Survey Validation and Optimization to Assess Public Awareness of Origin Protected Designation (PDO) Cheeses: Majorero and Palmero Cases
Source: Foods. 2026 Aug 18;15(16):2881. doi: 10.3390/foods15162881 (PMC13512008; doi:10.3390/foods15162881)
Supplement: Supplementary file 1 [file foods-15-02881-s001.zip › Material S1.pdf]

Material S1. Survey Questionnaire (English Translation)

Project: RTA2014-00047-C-00-00: *Valorization of traditional goat cheeses linked to autochthonous breeds. Traceability of milk origin using molecular markers and definition of new objective sensory quality parameters identifiable by consumers.*

Survey for participants (administered before and after training sessions)

Date:
Location:

Identification:

- Name (or anonymous code):
- Profession:
- Age:
- Sex:

Section 1. General Information

1. Do you like cheese? □ Yes □ No (Question no. 1)
2. Indicate your level of knowledge in the following areas:

- Canarian cheeses: Low / Medium / High (Question no. 2)
- Designations of Origin (PDO): Low / Medium / High (Question no. 3)
- Sensory analysis: Low / Medium / High (Question no. 4)

3. Have you ever attended a sensory analysis course? □ Yes □ No (Question no. 5)
    - If yes, for which product(s)? (Question no. 6)

Section 2. Sensory Attributes

Please rate how easy or difficult it is for you to identify the following attributes (1 = very difficult, 5 = very easy).

- **Visual / Texture attributes**
    - External appearance (Question no. 7)
    - Appearance when cut (Question no. 8)
    - Texture to touch (Question no. 9)
    - Roughness (Question no. 10)
    - Elasticity (Question no. 11)
    - Humidity (Question no. 12)
    - Texture in mouth (Question no. 13)
    - Firmness (Question no. 14)

  - Friability (Question no. 15)
  - Moisture in mouth (Question no. 16)
  - Adhesiveness (Question no. 17)
  - Solubility (Question no. 18)
  - Granularity / microstructure (Question no. 19)
- **Olfactory attributes**
  - Aroma (Question no. 20)
  - Intensity (Question no. 21)
  - Dairy family (Question no. 22)
  - Plant family (Question no. 23)
  - Fruity family (Question no. 24)
  - Roasted family (Question no. 25)
  - Spices family (Question no. 26)
  - Other families (defined by respondents and processed afterwards) (Question no. 27)
- **Taste attributes**
  - Salty (Question no. 28)
  - Sweet (Question no. 29)
  - Acidic (Question no. 30)
  - Bitter (Question no. 31)
  - Spicy (Question no. 32)
  - Astringent (Question no. 33)
  - Aftertaste (Question no. 34)
  - Persistence (Question no. 35)
  - Others (defined by respondents and processed afterwards) (Question no. 36)

Section 3. Knowledge of PDO Regulations

Mark each statement as True (T), False (F), or No Opinion/No Response (NONR).

- PDO regulations are the only mechanism that guarantee the hygienic–sanitary quality of cheeses. (Question no. 37)
- PDO regulations always add value (price) to the products. (Question no. 38)
- PDO regulations require production to always be ecological and sustainable. (Question no. 39)
- PDO regulations serve only to prevent fraud with equivalent products, not to generate added value. (Question no. 40)

- PDO regulations are requested at the initiative of the farmers. (Question no. 41)
- PDO regulations are always established at the initiative of the Administration. (Question no. 42)
- PDO regulations define the product as a Differentiated Quality Product. (Question no. 43)
- PDO regulations link the product to its territory. (Question no. 44)
- PDO regulations make compliance mandatory for farmers and cheesemakers producing cheese. (Question no. 45)
- PDO regulations require that production be linked to a breed. (Question no. 46)
- PDO regulations should link production to an autochthonous (native) breed. (Question no. 47)
- PDO regulations assign management to the producers. (Question no. 48)
- PDO regulations assign management to the Administration. (Question no. 49)
- PDO regulations state that it is sufficient for cheeses to be identified with a label. (Question no. 50)
- PDO regulations require cheeses to carry both a label and a numbered counter-label from the Regulatory Council. (Question no. 51)
- PDO regulations specify that the PDO name refers to a geographical area (in most cheeses) that the PDO protects. (Question no. 52)
- PDO regulations allow cheese to be produced outside the area that the PDO protects (defines). (Question no. 53)
- PDO regulations allow cheese to be matured outside the area that the PDO protects (defines). (Question no. 54)
- PDO regulations require that the area of production, elaboration, and maturation coincide with what the PDO defines and protects. (Question no. 55)
- PDO regulations allow animals to be housed in another area (municipality) different from those defined by the PDO. (Question no. 56)
- PDO regulations prohibit animals from grazing outside the municipalities (area) defined by the PDO. (Question no. 57)
- PDO regulations require cheesemaking technology to comply with the specifications of the Regulation. (Question no. 58)
- PDO regulations allow cheesemakers to coagulate at any temperature they consider appropriate. (Question no. 59)
- PDO regulations allow cheesemakers to use the rennet or coagulant they consider suitable. (Question no. 60)
- PDO regulations make cheesemakers responsible for the entire process of elaboration and labeling. (Question no. 61)
- PDO regulations allow the maturation of cheeses to be carried out outside the area (municipality) defined by the PDO. (Question no. 62)
- PDO regulations represent a European Union quality protection system. (Question no. 63)
- PDO regulations allow the use of milk from any species or breed. (Question no. 64)
- PDO regulations specify that only milk from the species listed in the Regulation may be used. (Question no. 65)

Section 4. PDO Majorero Cheese

Mark as T/F/NONR.

- Majorero PDO regulations allow cheese to be made with milk from any goat breed in the Canary Islands. (Question no. 66)

- Majorero PDO regulations require cheese to be made exclusively with Majorera goat milk. (Question no. 67)
- Majorero PDO regulations require cheese to be made with Majorera goat milk but allow up to 15% Canary sheep milk in cured cheeses. (Question no. 68)
- Majorero PDO regulations define Majorera goats as those born on the island of Fuerteventura. (Question no. 69)
- Majorero PDO regulations define Majorera goats as those that meet the breed standard and are registered in the herdbook. (Question no. 70)
- Majorero PDO regulations allow Majorero PDO cheese to be made with Majorera goat milk produced outside the island of Fuerteventura. (Question no. 71)
- Majorero PDO regulations allow cheese production in both Fuerteventura and Lanzarote. (Question no. 72)
- Majorero PDO regulations specify that cheese can only be produced in Fuerteventura. (Question no. 73)
- Majorero PDO regulations allow cheese maturation on another island. (Question no. 74)
- Majorero PDO regulations require all cheeses to carry a numbered counter-label with the PDO logo. (Question no. 75)
- Majorero PDO regulations allow fresh cheeses to be counter-labeled and protected. (Question no. 76)
- Majorero PDO regulations allow matured cheeses to be coated with paprika, gofio, or oil. (Question no. 77)
- Majorero PDO regulations require cheesemaking techniques to comply with the specifications in the PDO regulation. (Question no. 78)
- Majorero PDO regulations allow the use of vegetable coagulant in some cheeses. (Question no. 79)
- Majorero PDO regulations establish the breed as fundamental in this PDO. (Question no. 80)
- Majorero PDO regulations state that the breed is not important in this PDO. (Question no. 81)
- Majorero PDO regulations allow any cheese made on the island of Fuerteventura to be called “Queso Majorero.” (Question no. 82)
- Majorero PDO regulations state that cheeses at the point of sale do not need to carry a numbered counter-label; a label alone is sufficient. (Question no. 83)
- Majorero PDO regulations specify that only cheeses produced in dairies registered with the Regulatory Council of Majorero PDO can be called “Queso Majorero.” (Question no. 84)
- Majorero PDO regulations specify that only cheeses produced in dairies registered with the Regulatory Council of Majorero PDO and identified with its numbered counter-label can be called “Queso Majorero.” (Question no. 85)

Section 5. PDO Palmero Cheese

Mark as T/F/NONR.

- Palmero PDO regulations allow cheese to be made with Palmera goat milk and Palmera sheep milk. (Question no. 86)
- Palmero PDO regulations require cheese to be made exclusively with Palmera goat milk. (Question no. 87)
- Palmero PDO regulations define Palmera goats as those born on the island of La Palma. (Question no. 88)

- Palmero PDO regulations define Palmera goats as those that meet the breed standard and are registered in the herdbook. (Question no. 89)
- Palmero PDO regulations allow Palmero PDO cheese to be made with Palmera goat milk produced outside the island of La Palma. (Question no. 90)
- Palmero PDO regulations protect all municipalities on the island of La Palma. (Question no. 91)
- Palmero PDO regulations allow cheeses to be matured on another island. (Question no. 92)
- Palmero PDO regulations state that cheeses only need to carry one label. (Question no. 93)
- Palmero PDO regulations require cheeses to be identified with both a label and a numbered counter-label with the PDO logo. (Question no. 94)
- Palmero PDO regulations allow fresh cheeses to be counter-labeled and protected. (Question no. 95)
- Palmero PDO regulations allow cheeses to be coated with paprika. (Question no. 96)
- Palmero PDO regulations allow matured cheeses to be coated with oil, flour, or gofio. (Question no. 97)
- Palmero PDO regulations state that cheesemaking techniques do not need to comply with the specifications in the PDO regulation. (Question no. 98)
- Palmero PDO regulations allow the use of vegetable coagulant in some cheeses. (Question no. 99)
- Palmero PDO regulations establish the breed as fundamental in this PDO. (Question no. 100)
- Palmero PDO regulations state that the breed is not important in this PDO. (Question no. 101)
- Palmero PDO regulations allow any cheese made on the island of La Palma to be called "Queso Palmero." (Question no. 102)
- Palmero PDO regulations require cheeses at the point of sale to always be identified with both a label and a numbered PDO counter-label. (Question no. 103)
- Palmero PDO regulations specify that only cheeses produced in dairies registered with the Regulatory Council of Palmero PDO can be called "Queso Palmero." (Question no. 104)
- Palmero PDO regulations specify that only cheeses produced in dairies registered with the Regulatory Council of Palmero PDO and identified with its numbered counter-label can be called "Queso Palmero." (Question no. 105)

Section 6. Labeling Preferences

Which elements should differentiate Majorero and Palmero PDO cheeses on the label? Rank 1 (highest priority) to 5 (lowest):

- PDO regulations require that the label identify the island. (Question no. 106)
- PDO regulations require that the label identify the breed. (Question no. 107)
- PDO regulations require that the label indicate the feeding of the goats. (Question no. 108)
- PDO regulations require that the label identify the producer. (Question no. 109)
- PDO regulations require that the label indicate the method of production. (Question no. 110)
- PDO regulations allow other elements to be included on the label (specify). (Question no. 111)

- Add comments (e.g., What other elements should be included on the label). (Question no. 112)

End of survey.
Thank you for your collaboration. If you wish to receive results, please provide your email.

Legend

True (T), False (F), or No Opinion/No Response (NONR).

PDO: Protected Designation of Origin (PDO).
