# Supplementary material for: Survey Validation and Optimization to Assess Public Awareness of Origin Protected Designation (PDO) Cheeses: Majorero and Palmero Cases
Source: Foods. 2026 Aug 18;15(16):2881. doi: 10.3390/foods15162881 (PMC13512008; doi:10.3390/foods15162881)
Supplement: Supplementary file 1 [file foods-15-02881-s001.zip › Material S2.pdf]

Material S2. Post Hoc Power Analysis for Canonical Discriminant Analysis. R script used to estimate post hoc statistical power ($1 - \beta$) from the observed F statistics of the Canonical Discriminant Analysis using the non-central F distribution. The script calculates power for each conceptual domain evaluated in the questionnaire based on the representative F statistics and corresponding degrees of freedom.

```
################################################################
## Post Hoc Power Analysis for Canonical Discriminant Analysis
## Based on observed F statistics
## Compatible with R 4.5.x
################################################################

rm(list = ls())

alpha <- 0.05
N <- 530

# Assuming two groups (pre vs post)
df1 <- 1
df2 <- N - 2

################################################################
# Domains and largest observed F-values
################################################################

results <- data.frame(

Domain = c(
"Self-perceived knowledge",
"Sensory texture",
"Olfactory perception",
"Gustatory perception",
"PDO sanitary misconceptions",
"PDO governance",
"PDO territorial linkage",
"PDO labeling",
"PDO technological regulations",
"Milk-origin restrictions",
"Native breeds",
"Majorero commercialization",
"Palmero commercialization",
"Labeling preferences",
"Reduction in uncertainty",
"Stable variables"
),

F = c(
```

```
101.00,
199.66,
91.60,
66.67,
43.34,
22.31,
201.52,
135.11,
125.90,
304.20,
114.30,
81.33,
167.68,
7.49,
164.62,
2.67
)

)

#################################################################
# Function to calculate observed power
#################################################################

ObservedPower <- function(Fvalue, df1, df2, alpha = 0.05){

  lambda <- Fvalue * df1

  Fcrit <- qf(
    1-alpha,
    df1,
    df2
  )

  power <- 1-pf(
    Fcrit,
    df1,
    df2,
    ncp=lambda
  )

  return(power)

}

#################################################################
# Calculate power
#################################################################
```

```
results$Power <- mapply(
  ObservedPower,
  results$F,
  MoreArgs=list(
    df1=df1,
    df2=df2,
    alpha=alpha
  )
)

results$Power <- round(results$Power,6)

############################################################
# Print results
############################################################

print(results)

############################################################
# Summary
############################################################

cat("\n========================================\n")

cat("Minimum power :", round(min(results$Power),4),"\n")
cat("Mean power    :", round(mean(results$Power),4),"\n")
cat("Maximum power :", round(max(results$Power),4),"\n")

cat("========================================\n")
```
