# Supplementary material for: Survey Validation and Optimization to Assess Public Awareness of Origin Protected Designation (PDO) Cheeses: Majorero and Palmero Cases
Source: Foods. 2026 Aug 18;15(16):2881. doi: 10.3390/foods15162881 (PMC13512008; doi:10.3390/foods15162881)
Supplement: Supplementary file 1 [file foods-15-02881-s001.zip › Material S3.pdf]

Material S3. Discriminant Function 1 (F1).

F1 = 0.1556*Q7 - 0.0743*Q8 - 0.2818*Q9 + 0.2951*Q10 - 0.0352*Q11 - 0.0074*Q12 + 0.3005*Q13 - 0.1100*Q14 + 0.1362*Q15 + 0.1788*Q16 - 0.1786*Q17 - 0.0266*Q18 + 0.0928*Q19 - 0.0606*Q20 - 0.1027*Q21 + 0.2679*Q22 + 0.0560*Q23 - 0.2198*Q25 + 0.0094*Q26 - 0.0285*Q27 + 0.2229*Q28 - 0.1595*Q29 + 0.1208*Q30 - 0.3413*Q31 + 0.2244*Q32 + 0.0998*Q33 + 0.1698*Q3 Low + 0.3802*Q3 Medium - 0.2778*Q4 Low - 0.0796*Q4 Medium - 0.1248*Q5 No - 0.1185*Q WineYes + 0.0599*Q OilNo + 0.1102*Q BeerNo + 0.1072*Q ChocolateNo + 0.1479*Q FalseNo + 0.0811*Q MeatNo + 0.0699*Q HamNo - 0.0317*Q SparklingWineYes + 0.0158*Q LecheNo - 0.1241*Q36 False - 0.0496*Q36 NoOpinion + 0.1358*Q37 True + 0.0313*Q38 False - 0.0647*Q39 NoOpinion - 0.0994*Q39 False - 0.0819*Q40 False + 0.0688*Q40 NoOpinion + 0.0595*Q41 NoOpinion - 0.0427*Q42 False - 0.0159*Q42 True + 0.0270*Q43 True + 0.0805*Q44 False + 0.1058*Q44 NoOpinion + 0.0634*Q45 NoOpinion - 0.1553*Q45 False + 0.1086*Q47 False - 0.1416*Q47 True + 0.0715*Q49 True + 0.2712*Q49 False + 0.0352*Q51 True + 0.1241*Q51 False - 0.2435*Q53 NoOpinion - 0.0701*Q55 True - 0.1299*Q55 False - 0.1620*Q56 False - 0.0879*Q56 NoOpinion - 0.0816*Q57 NoOpinion + 0.1452*Q57 False - 0.1037*Q58 False + 0.0542*Q58 NoOpinion + 0.2234*Q59 NoOpinion + 0.0599*Q59 False - 0.0109*Q60 False - 0.0566*Q60 NoOpinion - 0.0431*Q62 True + 0.0187*Q62 False - 0.1087*Q63 True + 0.0094*Q63 NoOpinion + 0.5421*Q65 True + 0.0743*Q66 NoOpinion - 0.0655*Q66 True - 0.0322*Q68 True + 0.0437*Q68 False + 0.0443*Q69 True + 0.2108*Q69 False + 0.0641*Q70 NoOpinion - 0.1088*Q70 True - 0.0666*Q71 NoOpinion + 0.1126*Q71 False - 0.0582*Q73 True - 0.3680*Q73 NoOpinion + 0.0678*Q75 False + 0.1226*Q75 NoOpinion - 0.0737*Q76 True - 0.1813*Q76 NoOpinion - 0.2736*Q78 False + 0.0167*Q78 True - 0.0496*Q79 False + 0.0515*Q79 NoOpinion + 0.0146*Q80 True - 0.1330*Q80 NoOpinion + 0.1247*Q81 NoOpinion + 0.0281*Q81 False + 0.3210*Q82 True + 0.1564*Q82 NoOpinion - 0.0040*Q84 False + 0.0448*Q84 True + 0.0613*Q85 False + 0.0765*Q85 NoOpinion - 0.0103*Q86 True + 0.0209*Q86 NoOpinion + 0.0144*Q88 NoOpinion - 0.0264*Q88 False - 0.0762*Q89 NoOpinion - 0.0322*Q89 True + 0.0472*Q92 NoOpinion - 0.0545*Q92 False + 0.0014*Q94 True + 0.0572*Q94 NoOpinion + 0.0500*Q95 False - 0.0024*Q95 NoOpinion + 0.1333*Q101 True + 0.1673*Q101 False - 0.0397*Q104 False + 0.0309*Q104 NoOpinion - 0.0166*Q106 False - 0.1984*Q106 NoOpinion + 0.0601*Q111 2 - 0.1870*Q111 1 + 0.1055*Q111 4 - 0.0346*Q111 NoOpinion - 0.1638*Q111 3 - 0.1702*Q112 3 - 0.0643*Q112 1 - 0.1974*Q112 2 - 0.0093*Q112 5
