# Supplementary material for: Survey Validation and Optimization to Assess Public Awareness of Origin Protected Designation (PDO) Cheeses: Majorero and Palmero Cases
Source: Foods. 2026 Aug 18;15(16):2881. doi: 10.3390/foods15162881 (PMC13512008; doi:10.3390/foods15162881)
Supplement: Supplementary file 1 [file foods-15-02881-s001.zip › Table S3.pdf]

Table S3. Results for the tests of equality of group means to test for difference in the means depending on evaluation moment (before or after training).

Question	Wilks' Lambda	F	DF1	DF2	p-value
65-True	0.6345	304.2002	1	528	< 0.0001
71-False	0.7238	201.5248	1	528	< 0.0001
13	0.7256	199.6597	1	528	< 0.0001
96-False	0.7590	167.6771	1	528	< 0.0001
73-No Opinion - No Response	0.7623	164.6187	1	528	< 0.0001
16	0.7730	155.0736	1	528	< 0.0001
15	0.7944	136.6483	1	528	< 0.0001
51-False	0.7962	135.1086	1	528	< 0.0001
76-No Opinion - No Response	0.8033	129.2698	1	528	< 0.0001
71-No Opinion - No Response	0.8068	126.4544	1	528	< 0.0001
59-False	0.8075	125.8960	1	528	< 0.0001
92-No Opinion - No Response	0.8180	117.4962	1	528	< 0.0001
68-False	0.8206	115.4251	1	528	< 0.0001
89-No Opinion - No Response	0.8220	114.3049	1	528	< 0.0001
69-False	0.8330	105.8737	1	528	< 0.0001
4-Low	0.8394	100.9956	1	528	< 0.0001
88-No Opinion - No Response	0.8424	98.8015	1	528	< 0.0001
53-No Opinion - No Response	0.8478	94.7828	1	528	< 0.0001
59-No Opinion - No Response	0.8485	94.2428	1	528	< 0.0001
3-Low	0.8515	92.0972	1	528	< 0.0001
80-No Opinion - No Response	0.8520	91.6992	1	528	< 0.0001
22	0.8522	91.6020	1	528	< 0.0001
53-False	0.8567	88.3050	1	528	< 0.0001
70-True	0.8593	86.4258	1	528	< 0.0001
19	0.8614	84.9240	1	528	< 0.0001
82-True	0.8665	81.3253	1	528	< 0.0001
4-Medium	0.8669	81.0457	1	528	< 0.0001
17	0.8694	79.3148	1	528	< 0.0001
10	0.8834	69.6986	1	528	< 0.0001
84-True	0.8845	68.9662	1	528	< 0.0001
30	0.8879	66.6726	1	528	< 0.0001
82-No Opinion - No Response	0.8880	66.5827	1	528	< 0.0001
14	0.8891	65.8371	1	528	< 0.0001

Question	Wilks' Lambda	F	DF1	DF2	p-value
45-No Opinion - No Response	0.8904	64.9813	1	528	< 0.0001
5-No	0.8927	63.4845	1	528	< 0.0001
20	0.8936	62.8442	1	528	< 0.0001
21	0.8976	60.2456	1	528	< 0.0001
70-No Opinion - No Response	0.8983	59.8049	1	528	< 0.0001
81-No Opinion - No Response	0.9025	57.0721	1	528	< 0.0001
47-False	0.9048	55.5222	1	528	< 0.0001
3-Medium	0.9060	54.7658	1	528	< 0.0001
101-No Opinion - No Response	0.9081	53.4072	1	528	< 0.0001
32	0.9089	52.9060	1	528	< 0.0001
11	0.9136	49.9491	1	528	< 0.0001
79-False	0.9145	49.3455	1	528	< 0.0001
23	0.9156	48.6814	1	528	< 0.0001
43-True	0.9194	46.2685	1	528	< 0.0001
38-True	0.9241	43.3393	1	528	< 0.0001
57-No Opinion - No Response	0.9262	42.0638	1	528	< 0.0001
12	0.9282	40.8330	1	528	< 0.0001
39-No Opinion - No Response	0.9307	39.3261	1	528	< 0.0001
94-No Opinion - No Response	0.9311	39.0416	1	528	< 0.0001
60-No Opinion - No Response	0.9322	38.4093	1	528	< 0.0001
79-No Opinion - No Response	0.9340	37.2985	1	528	< 0.0001
62-True	0.9343	37.1576	1	528	< 0.0001
44-No Opinion - No Response	0.9351	36.6506	1	528	< 0.0001
9	0.9388	34.4263	1	528	< 0.0001
31	0.9418	32.6178	1	528	< 0.0001
8	0.9423	32.3606	1	528	< 0.0001
37-True	0.9473	29.3447	1	528	< 0.0001
Cheese-Yes	0.9476	29.2148	1	528	< 0.0001
99-False	0.9476	29.2074	1	528	< 0.0001
86-No Opinion - No Response	0.9510	27.2261	1	528	< 0.0001
57-False	0.9513	27.0215	1	528	< 0.0001
95-No Opinion - No Response	0.9519	26.7021	1	528	< 0.0001
7	0.9533	25.8603	1	528	< 0.0001
58-No Opinion - No Response	0.9533	25.8388	1	528	< 0.0001

Question	Wilks' Lambda	F	DF1	DF2	p-value
99-No Opinion - No Response	0.9543	25.2884	1	528	< 0.0001
39-False	0.9553	24.6942	1	528	< 0.0001
94-True	0.9594	22.3588	1	528	< 0.0001
49-True	0.9595	22.3053	1	528	< 0.0001
85-False	0.9602	21.8904	1	528	< 0.0001
66-No Opinion - No Response	0.9613	21.2624	1	528	< 0.0001
63-No Opinion - No Response	0.9619	20.8966	1	528	< 0.0001
78-False	0.9628	20.4093	1	528	< 0.0001
86-True	0.9628	20.3858	1	528	< 0.0001
27	0.9638	19.8333	1	528	< 0.0001
55-True	0.9667	18.2063	1	528	< 0.0001
85-No Opinion - No Response	0.9679	17.5113	1	528	< 0.0001
101-False	0.9722	15.1258	1	528	0.0001
81-False	0.9722	15.0925	1	528	0.0001
75-No Opinion - No Response	0.9755	13.2612	1	528	0.0003
63-True	0.9765	12.7168	1	528	0.0004
95-False	0.9766	12.6388	1	528	0.0004
56-No Opinion - No Response	0.9768	12.5183	1	528	0.0004
68-True	0.9786	11.5705	1	528	0.0007
69-True	0.9791	11.2896	1	528	0.0008
36-No Opinion - No Response	0.9808	10.3589	1	528	0.0014
51-True	0.9824	9.4631	1	528	0.0022
58-False	0.9828	9.2425	1	528	0.0025
65-False	0.9852	7.9061	1	528	0.0051
28	0.9853	7.8962	1	528	0.0051
56-False	0.9854	7.8428	1	528	0.0053
88-False	0.9856	7.7119	1	528	0.0057
41-No Opinion - No Response	0.9858	7.5804	1	528	0.0061
107-2	0.9860	7.4865	1	528	0.0064
89-True	0.9861	7.4279	1	528	0.0066
92-False	0.9864	7.3018	1	528	0.0071
18	0.9864	7.2600	1	528	0.0073
49-False	0.9874	6.7191	1	528	0.0098
40-No Opinion - No Response	0.9879	6.4652	1	528	0.0113
47-True	0.9906	5.0301	1	528	0.0253
42-True	0.9921	4.2118	1	528	0.0406
66-True	0.9924	4.0420	1	528	0.0449
73-True	0.9932	3.6107	1	528	0.0580

Question		Wilks' Lambda	F	DF1	DF2	p-value
107-5		0.9935	3.4383	1	528	0.0643
	33	0.9950	2.6701	1	528	0.1028
106-4		0.9954	2.4158	1	528	0.1207
106-3		0.9959	2.1584	1	528	0.1424
76-True		0.9961	2.0893	1	528	0.1489
60-False		0.9963	1.9767	1	528	0.1603
106-2		0.9977	1.2369	1	528	0.2666
107-3		0.9977	1.2298	1	528	0.2679
106-1		0.9978	1.1477	1	528	0.2845
107-1		0.9982	0.9705	1	528	0.3250
	26	0.9983	0.8769	1	528	0.3495
	25	0.9986	0.7258	1	528	0.3946
40-False		0.9989	0.5962	1	528	0.4404
84-False		0.9990	0.5466	1	528	0.4601
96-True		0.9990	0.5287	1	528	0.4675
42-False		0.9992	0.4310	1	528	0.5118
36-False		0.9994	0.3240	1	528	0.5695
45-False		0.9994	0.3051	1	528	0.5809
41-False		0.9995	0.2773	1	528	0.5987
38-False		0.9995	0.2759	1	528	0.5996
62-False		0.9996	0.2245	1	528	0.6358
37-False		0.9997	0.1833	1	528	0.6688
80-True		0.9997	0.1576	1	528	0.6915
75-False		0.9998	0.0865	1	528	0.7688
44-False		0.9999	0.0771	1	528	0.7814
Oil-No		0.9999	0.0535	1	528	0.8172
	29	0.9999	0.0451	1	528	0.8319
78-True		0.9999	0.0341	1	528	0.8536
55-False		0.9999	0.0314	1	528	0.8595
Wine-Yes		0.9999	0.0275	1	528	0.8684
43-False		1.0000	0.0019	1	528	0.9649
106-No Opinion - No Response		1.0000	0.0013	1	528	0.9713
Chocolate-No		1.0000	0.0001	1	528	0.9914
Fish-No		1.0000	0.0001	1	528	0.9926
Coffee-No		1.0000	0.0001	1	528	0.9926
Beer-No		1.0000	0.0001	1	528	0.9940
Honey-No		1.0000	0.0001	1	528	0.9940
Ham-No		1.0000	0.0001	1	528	0.9940
Milk-No		1.0000	0.0001	1	528	0.9940
Carne-No		1.0000	0.0000	1	528	0.9957
Sparkling Wine-Yes		1.0000	0.0000	1	528	0.9957
3-High				1	528	
4-High				1	528	
5-Yes				1	528	
Cheese-No				1	528	

Question	Wilks' Lambda	F	DF1	DF2	p-value
Wine-No			1	528	
Oil-Yes			1	528	
Beer-Yes			1	528	
Chocolate-Yes			1	528	
Honey-Yes			1	528	
Fish-Yes			1	528	
Carne-Yes			1	528	
Coffee-Yes			1	528	
Ham-Yes			1	528	
Sparkling Wine-No			1	528	
Milk-Yes			1	528	
36-True			1	528	
37-No Opinion - No Response			1	528	
38-No Opinion - No Response			1	528	
39-True			1	528	
40-True			1	528	
41-True			1	528	
42-No Opinion - No Response			1	528	
43-No Opinion - No Response			1	528	
44-True			1	528	
45-True			1	528	
47-No Opinion - No Response			1	528	
49-No Opinion - No Response			1	528	
51-No Opinion - No Response			1	528	
53-True			1	528	
55-No Opinion - No Response			1	528	
56-True			1	528	
57-True			1	528	
58-True			1	528	
59-True			1	528	
60-True			1	528	
62-No Opinion - No Response			1	528	
63-False			1	528	
65-No Opinion - No Response			1	528	
66-False			1	528	
68-No Opinion - No Response			1	528	
69-No Opinion - No Response			1	528	
70-False			1	528	
71-True			1	528	
73-False			1	528	
75-True			1	528	
76-False			1	528	
78-No Opinion - No Response			1	528	
79-True			1	528	
80-False			1	528	
81-True			1	528	

Question	Wilks' Lambda	F	DF1	DF2	p-value
82-False			1	528	
84-No Opinion - No Response			1	528	
85-True			1	528	
86-False			1	528	
88-True			1	528	
89-False			1	528	
92-True			1	528	
94-False			1	528	
95-True			1	528	
96-No Opinion - No Response			1	528	
99-True			1	528	
101-True			1	528	
106-5			1	528	
107-4			1	528	
107-No Opinion - No Response			1	528	
